# AI-Based Clinical Decision Support Systems in Primary Care: Systematic Review of Clinical Implementation

**DOI:** 10.2196/96882

**Published:** 2026-10-19

**Authors:** Florian Naye, Steven Ouellet, Farzaneh Yousefi, Chloé Cachinho, Frédéric Bergeron, Onil Bhattacharyya, Vess Stamenova, Maxime Sasseville

**Affiliations:** 1 Faculty of Nursing Sciences, Université Laval, 1050, avenue de la Médecine, Québec, QC, G1V 0A6, Canada, 1 418-290-3089; 2 VITAM Research Center on Sustainable Healt, Québec, QC, Canada; 3 Bibliothèque de l’Université Laval, Université Laval, Québec, QC, Canada; 4 Centre for Digital Health Evaluation, Women's College Hospital, Toronto, QC, Canada; 5 Department of Family and Community Medicine, University of Toronto, Toronto, ON, Canada; 6 Department of Information Technology Management, Toronto Metropolitan University, Toronto, ON, Canada

**Keywords:** artificial intelligence, clinical decision support systems, primary health care, systematic review, patient safety, effectiveness, implementation, Preferred Reporting Items for Systematic Reviews and Meta-Analyses, PRISMA

## Abstract

**Background:**

AI-based clinical decision support systems (AI-CDSSs) can help primary care clinicians provide higher quality care amid rising case complexity and resource constraints. However, evidence regarding their effectiveness and safety in primary care practice is lacking.

**Objective:**

We synthesized evidence on the effectiveness and safety of AI-CDSSs compared with usual care or non–AI-based systems in primary care settings.

**Methods:**

We conducted a systematic review following the Cochrane Handbook for Systematic Reviews of Interventions and reported the results according to PRISMA (Preferred Reporting Items for Systematic Reviews and Meta-Analyses) 2020 guidelines. We included experimental and quasi-experimental studies evaluating AI-CDSSs used by health care professionals in primary care settings, compared with usual care or non–AI-based decision support systems, and that reported clinician-, patient-, or system-level outcomes, or safety. An information specialist designed the search strategies and systematically reviewed MEDLINE, Embase, CINAHL, Web of Science, and CENTRAL from inception to June 27, 2025. Reviewers independently performed the selection and extraction processes. Data extraction was informed by the DECIDE-AI and CONSORT-AI (Consolidated Standards of Reporting Trials–AI) reporting guidelines and the APPRAISE-AI tool. We independently assessed the risk of bias using Risk of Bias-2 or Risk of Bias in Non-Randomized Studies of Interventions. Owing to substantial heterogeneity, we conducted a synthesis without meta-analysis and explored heterogeneity using an adapted Non-Adoption, Abandonment, Scale-Up, Spread, and Sustainability–informed implementation-context classification. The review protocol was registered in PROSPERO (CRD420251119225).

**Results:**

We identified 4074 records through database searches and 12 additional records through citation searching and trial registration checking. We included 14 records on 11 distinct studies, including 4 experimental and 7 quasi-experimental studies. The risk of bias was high in all experimental studies and serious to critical in quasi-experimental studies. AI-CDSSs showed more consistent positive effects on workflow-related outcomes in structured and protocolized decision contexts, particularly for screening, referral, and alert optimization tasks. Patient-level effects were limited and inconsistent, particularly in more complex contexts involving heterogeneous conditions or preference-sensitive decisions. System-level outcomes were heterogeneous, with isolated improvements in service delivery and utilization outcomes. Safety evaluation was rare and poorly specified. Only 2 studies prespecified safety outcomes, and the reported safety information mainly involved technical malfunctions or self-reported side effects. No study reported clinician interaction measures such as agreement with, override of, or reliance on AI outputs. Interpretation of the findings is limited by substantial heterogeneity, short follow-up periods, predominantly high or critical risk of bias, and evidence derived exclusively from high-income countries.

**Conclusions:**

AI-CDSSs in primary care may provide more consistent benefits for workflow-related and process-level outcomes in structured and protocolized decision contexts, while evidence on safety remains insufficient to support confident routine implementation. This systematic review reveals an important gap between the rapid development of AI-CDSSs and the lack of robust, comparable evidence to support their reliable use in primary care.

## Introduction

Primary care is central to high-performing health systems, contributing to improved population health outcomes, greater equity, and reduced avoidable hospitalizations [1-4]. Yet, primary care is increasingly strained [5], with rising case complexity, workforce shortages, and fragmented information systems placing increasing pressure on clinicians [6-9]. At the same time, the volume of clinical evidence continues to expand at a pace that exceeds clinicians’ capacity to remain aligned with current best practices [10]. These challenges have accelerated interest in digital tools to support clinical decision-making and workflow in primary care settings [11,12].

Clinical decision support systems (CDSSs) can assist clinicians by generating patient-specific recommendations at the point of care [13]. Meta-analyses of controlled trials indicate that traditional CDSSs achieve small to moderate improvements in targeted processes of care (eg, prescribing, test ordering, documentation, and vaccination) [14]. However, these effects do not consistently translate into better clinical outcomes, with evidence indicating small and inconsistent improvements at the patient level [14]. This gap highlights a key limitation of traditional CDSSs, as they have limited capacity to account for variability in patient characteristics and clinical contexts [15,16]. AI methods can be integrated into CDSSs to improve data-driven precision and enable more individualized recommendations [15,16].

Most evidence on AI-based CDSSs (AI-CDSSs) remains concentrated in the early stages of the evaluation pathway [17-19]. Most AI-CDSS studies focus on model development and validation, often reporting strong technical performance [20-24]. Performance evaluations are typically conducted using curated datasets and predefined tasks that do not reflect the complexity and variability of routine clinical care [6,25,26]. Performance assessments conducted in closed contexts therefore poorly predict how AI-CDSSs will perform when deployed in clinical practice.

To be effective, AI-CDSSs must align with clinical workflows and support, rather than disrupt, the work of clinicians [7,27]. When there is a misalignment, decision support can introduce additional clinical or administrative steps into already time-constrained consultations or generate recommendations that are difficult to implement [28]. Clinical misalignments commonly lead to nonadoption, inappropriate use, and long-term abandonment of AI-CDSSs [7,29-31]. Even systems with strong technical performance have failed to produce clinical impact because they were not effectively integrated into clinical practice [32].

AI-CDSSs influence clinical decision-making in ways that shape diagnosis and management, raising clinical safety considerations [33]. Errors in predictions or recommendations, if not identified or appropriately contextualized, can lead to inappropriate or delayed care [34,35]. How clinicians interact with AI-CDSSs, such as relying on recommendations without sufficient critical appraisal or disregarding relevant outputs, can shape how these risks materialize in practice [36]. Data from AI incident databases document thousands of AI-related incidents and hazards reported over the past decade, emphasizing the scale of these concerns [37]. However, the processes through which risks are identified, monitored, and mitigated in clinical settings remain insufficiently characterized [37-39], limiting the ability to determine whether AI-CDSSs can be safely integrated into care.

The rapid development, increasing availability, and commercialization of AI-CDSSs are accelerating their clinical use [40,41]. This pace of deployment could exceed the availability of effectiveness and safety evidence in clinical settings [19,42-47]. When evaluation does not keep pace with deployment, systems are introduced into care without adequate evaluation in routine practice [45,48,49], limiting impact assessment on clinical decision-making and health system oversight [43,50]. Previous reviews have mapped AI use in primary care [24,51] and highlighted barriers to AI-CDSS implementation, including workflow integration and feasibility [52,53], but have not systematically synthesized whether AI-CDSSs improve outcomes or how safe they are compared with usual care in routine primary care settings. This evidence gap aligns with recent calls for clinical evaluation of AI-CDSSs to support implementation decisions in health care [41,44,45,54,55]. The aim of this review was to synthesize evidence on the effectiveness and safety of AI-CDSSs compared with usual care or non–AI-based systems in primary care settings.

## Methods

### Study Design

We conducted a systematic review following the Cochrane Handbook for Systematic Reviews of Interventions [56] and reported the results according to PRISMA (Preferred Reporting Items for Systematic Reviews and Meta-Analyses) 2020 guidelines [57]. The completed PRISMA 2020 checklist is provided in Checklist 1. We registered the protocol in PROSPERO [58]. Following protocol registration, departures from the registered methods were limited and are summarized in Multimedia Appendix 1. These consisted of planned analyses that were not undertaken because the characteristics of the included evidence did not support their meaningful application (Risk of Bias due to Missing Evidence and Grading of Recommendations Assessment, Development, and Evaluation [GRADE]), and 1 additional exploratory analysis introduced post hoc to facilitate interpretation of heterogeneity by classifying implementation contexts using an operationalized approach informed by the Non-Adoption, Abandonment, Scale-Up, Spread, and Sustainability (NASSS) framework.

### Eligibility Criteria

We present the eligibility criteria according to the population, intervention, comparator, and outcomes (PICO) framework [59] in Table 1.

**Table 1.** Eligibility criteria based on the PICO^a^ framework.

Category	Eligibility criteria
Population	Health care professionals (physicians, nurses, and allied health) or clinical teams. Exclusion: only students.
Intervention	AI-CDSS^b^. CDSSs are commonly defined as “computer systems designed to impact clinician decision making about individual patients at the point in time that these decisions are made” [13]. When CDSSs incorporate AI methods such as machine learning, they are referred to as AI-CDSS. For this synthesis, we defined AI-CDSSs as systems meeting all of the following criteria: (1) generates patient-specific outputs, (2) produces decision-directed outputs (including recommendation, prioritization, or optimization), (3) is used at or near the point of care within clinical workflow, (4) is clinician-facing, and (5) incorporates AI-based methods. Exclusion: presence of co-interventions (ie, additional interventions delivered alongside the AI-CDSS).
Comparators	Standard care with or without traditional (non-AI) CDSS.
Outcomes	Any quantitative outcomes at the patient level (eg, mortality and readmission), clinician level (eg, adherence and satisfaction), or system level (eg, cost and workflow). Adverse outcomes and unintended consequences.
Study designs	Randomized controlled trials, nonrandomized controlled trials, mixed methods studies, prospective cohort studies, and retrospective cohort studies with AI-CDSS clinical integration. Exclusion: retrospective cohort studies without AI-CDSS clinical integration, reviews, editorials, conference abstracts, preprints, and protocols.
Setting	Primary care setting.
Language of publication	English and French.

a PICO: population, intervention, comparator, and outcomes.

b AI-CDSS: AI-based clinical decision support system.

### Information Sources and Search Strategy

We developed a search strategy in collaboration with an experienced librarian (FB) and reported it in accordance with the PRISMA-S (Preferred Reporting Items for Systematic Reviews and Meta-Analyses literature search extension) guidelines [60] (Checklist 2). We tested the sensitivity of the strategy [61] using studies identified in a previous scoping review [51] that were expected to be retrieved by our strategy. The search was not updated after the initial sensitivity testing. We provide the full electronic search strategies for all databases in Multimedia Appendix 2.

We searched the following databases on June 27, 2025, without validated filters or date and language restrictions: MEDLINE (Ovid), Embase (Elsevier), CINAHL (EBSCOhost), Web of Science Core Collection (Clarivate), and the CENTRAL. We combined database-specific controlled vocabulary (eg, MeSH and Emtree) and free-text terms related to AI and CDSS with terms capturing study designs relevant to effectiveness and safety (ie, randomized, quasi-experimental, and observational studies). We did not search for gray literature sources (eg, conference proceedings and institutional reports) because of the focus on peer-reviewed evidence. However, we searched the trial registries of included studies to identify ongoing or unpublished results. We conducted backward and forward citation searching [62] of all included studies and relevant reviews identified during the selection process, using Scopus on August 6, 2025.

All retrieved records were imported into the management software Covidence (Veritas Health Innovation), where duplicates were removed using its automated function.

### Selection and Data Collection Processes

We conducted calibration training on eligibility criteria interpretation among reviewers (FN, MS, and SO) using the first 100 references identified. If interrater agreement (percent agreement) was below 80% [63], we clarified the eligibility criteria through discussion and a think-aloud approach [64] and conducted a new training. Pairs of reviewers (FN, MS, and SO) applied the eligibility criteria to independently screen the titles and abstracts of all potentially eligible citations and then to full-text articles retrieved. We resolved discrepancies by team consensus. We documented reasons for full-text exclusion and reported them using the PRISMA 2020 flow diagram.

### Data Items

We developed a standardized data extraction form informed by existing reporting guidelines, including DECIDE-AI for early-stage clinical evaluation of AI-CDSSs [65], CONSORT-AI (Consolidated Standards of Reporting Trials–AI) for clinical trials of AI interventions [66], and the APPRAISE-AI tool for quantitative evaluation of AI-CDSS studies [67]. Given the small number of eligible studies, the form was pilot tested on the most detailed study [68]. Two independent reviewers (FN and SO) performed the data extraction, and disagreements were resolved by a third reviewer (MS). Extracted variables covered study characteristics (eg, study period and number of participating institutions), participant characteristics (eg, sample size and baseline features), and intervention details (eg, purpose and outputs). We also collected data on outcomes and effects (eg, measurement instruments and between-group comparisons) and safety (eg, risk mitigation strategies and reported harms or unintended effects).

### Risk of Bias Assessment

We assessed individually randomized and cluster-randomized trials using the corresponding versions of Risk of Bias-2 [69] and quasi-experimental studies using ROBINS-I (version 2) [70]. Two reviewers (FN and SO) independently evaluated the risk of bias for the main outcome of each study, and disagreements were resolved by team consensus.

### Synthesis Method

Owing to substantial heterogeneity across studies, we did not conduct a meta-analysis [71,72]. First, there was limited evidence for any given comparison, with most outcomes reported in only 1 or 2 studies, resulting in minimal overlap of comparable effect estimates [72]. Second, studies exhibited marked clinical and methodological diversity, including variation in target conditions, AI-CDSS functions, implementation contexts, study designs, and outcome definitions [72]. Under these conditions, the assumption of a common underlying effect was not met, and quantitative pooling would risk producing misleading summary estimates. This approach is consistent with methodological guidance from the Cochrane Handbook [72].

We therefore performed a narrative synthesis and reported it in accordance with the Synthesis Without Meta-analysis (SWiM) reporting guidelines [71]. We categorized outcomes into 3 levels: patient level, referring to measures affecting patients, such as symptom improvement or function; clinician level, including outcomes related to provider knowledge, behavior, or workload; and system level, encompassing organizational or economic outcomes, such as health care utilization or cost-effectiveness. We synthesized findings using the direction of effects (improvement, no effect, or worsening), which was determined based on the statistical results reported in the original studies (eg, *P* values or equivalent information). All included studies were considered, and findings were interpreted using study design and risk of bias. We presented findings using structured tables and narrative summaries. We provide the completed SWiM checklist in Checklist 3.

We explored heterogeneity through structured comparison across implementation contexts. To describe the implementation context, we classified each study as simple, complicated, or complex using an operationalized approach informed by selected items from the NASSS-Complexity Assessment Tool (CAT) short version [73,74]. As NASSS-CAT was developed to support prospective assessment of technology projects and includes domains not consistently reported in published studies, we adapted relevant items into 4 observable criteria derived from the extracted data: condition complexity, decision logic, system complexity, and preference sensitivity. Each criterion was coded using predefined definitions (Multimedia Appendix 3). Two reviewers (FN and MS) independently classified context as *simple* when all criteria indicated low complexity and as *complex* when at least two criteria indicated high complexity. All other scenarios were classified as *complicated*.

We classified safety information according to the DECIDE-AI reporting guidelines [65] across 3 domains: patient safety (prespecification and reported harms or unintended effects), AI system (risk mitigation and significant errors or malfunctions), and clinician interaction (agreement and override).

For AI-CDSS functions reporting, we used the taxonomy of front-end clinical decision support tools [75]. We then complemented this classification by extracting the corresponding operational functions, defined as the concrete actions performed by the system within the study context (eg, alert optimization, monitoring and follow-up, treatment optimization, and documentation or care summary).

## Results

### Study Selection

We retrieved 4074 records from the search. After removing duplicates, 3034 (74.5%) records remained for the selection. Of these records, 126 (4.2%) were retained for full-text screening. We identified 12 supplemental records from handsearching and trial registration checking. At the end of the process, we included 14 records on 11 distinct studies (we present the mapping of included records to unique studies in Multimedia Appendix 4). We present a flow diagram of the selection process in Figure 1.

**Figure 1.**
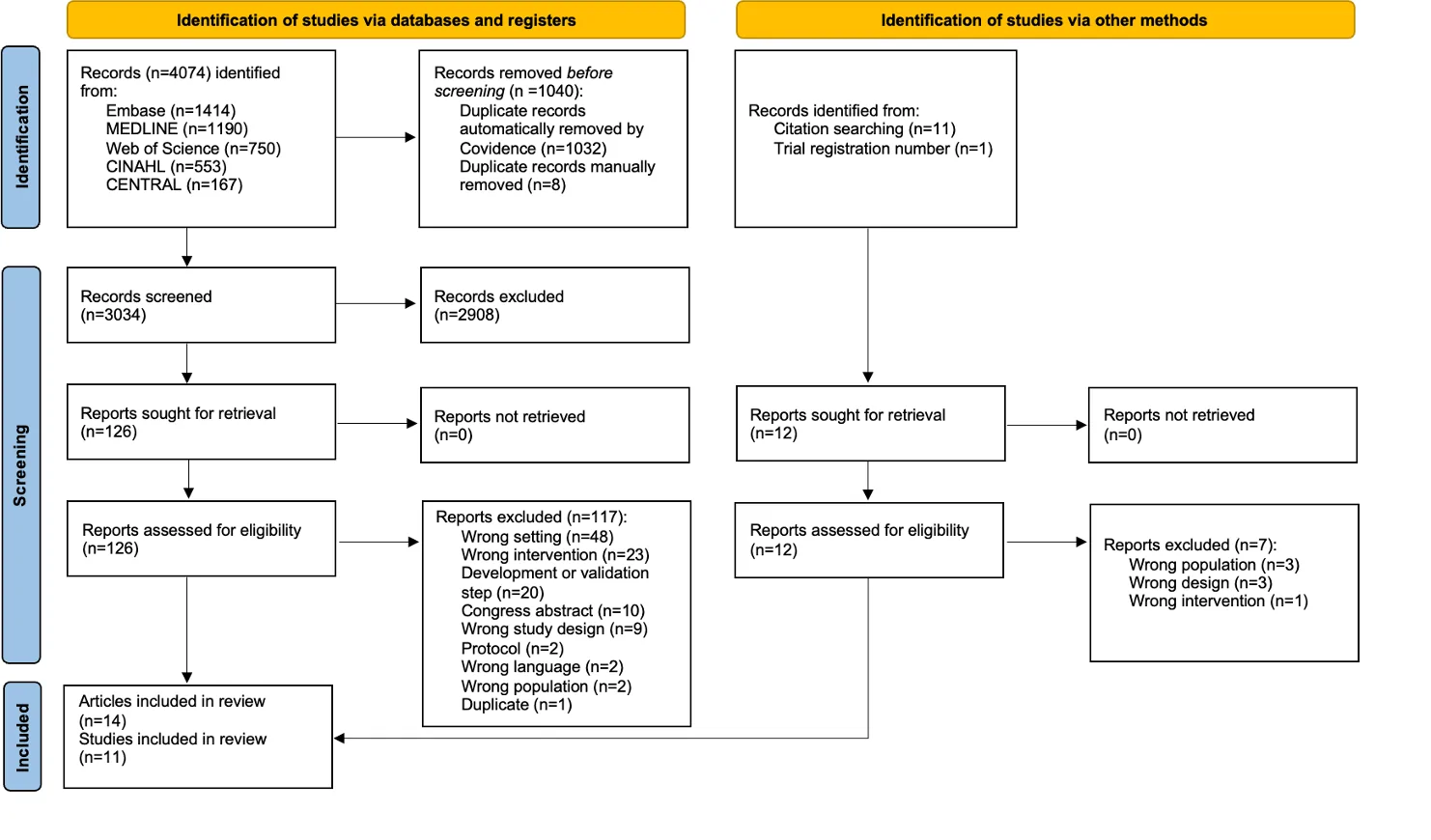
Flow diagram based on PRISMA 2020.

### Characteristics of the Studies

We present a summary of the characteristics of the included studies in Table 2, and a detailed version is available in Multimedia Appendix 5.

We identified 11 included studies published between 2019 and 2025 and conducted across North America (n=7, 63.6%) and Europe (n=4, 36.4%). Four (36.4%) studies used experimental designs, and 7 (63.6%) were quasi-experimental, applying a variety of comparison strategies, including concurrent control groups, historical or matched controls, and before-after or self-controlled analyses. Study durations ranged from approximately 1 to 12 months. According to the DECIDE-AI reporting items, none of the studies reported patient involvement in the evaluation of the interventions. Five (45.5%) studies reported at least one conflict of interest, and 5 (45.5%) were funded by industry.

**Table 2.** Characteristics of included studies evaluating AI-based clinical decision support systems in primary care, including level of evidence, type of control group, study period, country of recruitment, and reporting of conflicts of interest and funding sources.

Author	Year of publication	Level of evidence	Study period	Country of recruitment	Conflicts of interest	Sources of funding
Alcoceba-Herrero et al [76]	2024	Experimental study	~2 mo	Spain	No	Academic
Areias et al^a^ [77]	2024	Quasi-experimental study with concurrent control group	Control: ~24 mo; experimental: ~6 mo	United States	Yes	Industrial and governmental
Chen et al [78]	2020	Quasi-experimental study with alternating on and off periods	~2 mo	United States	No	Not reported
Cruz et al [79]	2019	Quasi-experimental before-after study	Control: ~16 mo; experimental: ~1 mo	Spain	Not reported	Not reported
Granviken et al [68]	2025	Experimental study	~9 mo	Norway	No	Governmental
Herter et al [80]	2022	Quasi-experimental study with matched control group	~4 mo	Netherlands	Yes	Industrial
Liu et al [81]	2021	Quasi-experimental study with historical control group	~8 mo	United States	Yes	Industrial
Popescu et al [82]	2021	Quasi-experimental study with self-control	~10 mo	Canada	Yes	Industrial
Qassim et al [83]	2023	Not reported	Not reported	Not reported	Not reported	Governmental
Romero-Brufau et al [84]	2020	Quasi-experimental pre-post survey-based quality improvement study	Control: ~1 mo; experimental: ~1 mo	United States	No	Academic
Seol et al [85]	2021	Experimental study	~12 mo	United States	No	Industrial and governmental
Yao et al [86]	2021	Experimental study	~8 mo	United States	Yes	Academic
Rushlow et al^a^ [87]	2022	Not reported	Not reported	Not reported	Not reported	Not reported
Thao et al^a^ [88]	2024	Not reported	Not reported	Not reported	Not reported	Not reported

a Post hoc analysis.

### Risk of Bias Assessment

We evaluated the overall risk of bias as high for all experimental studies. The main concern for the 2 parallel randomized controlled trials (RCTs) was related to deviations from the intended interventions. For the 2 cluster RCTs, the main concerns were linked to the identification and timing of recruitment and the measurement of the outcomes.

Among quasi-experimental studies, 1 was at serious risk of bias, while all others were at critical risk of bias. Five critical-level assessments were derived from the ROBINS-I screening stage (B1-B3), reflecting concerns related to uncontrolled confounding and/or outcome measurement. In the remaining study rated at critical risk of bias, concerns were related to missing data. We provide overall judgment and detailed domain-level assessments in Multimedia Appendix 6.

### Characteristics of the Participants

We present a summary of the characteristics of the participants included in the studies in Table 3, and a detailed version is presented in Multimedia Appendix 7.

AI-CDSSs were primarily evaluated in musculoskeletal conditions (3/11, 27.3%), urinary tract infections (2/11, 18.2%), and diabetes (2/11, 18.2%), with 1 (9.1%) study addressing multiple conditions. Of the 11 studies, 6 (54.5%) reported clinician sample sizes, ranging from 7 to 358. Among these 6 studies, 4 (66.7%) did not report any clinician characteristics. Age and sex were reported in 2 (33.3%) studies, and ethnicity in 1 (16.7%) study. Of the 11 studies, 8 (72.7%) reported patient sample sizes, ranging from 14 to 24,083 in the experimental groups and from 14 to 12,103 in the control groups. Among these 8 studies, age was reported in all (100%), sex and ethnicity in 5 (62.5%), and gender in 4 (50%). When reported (8/11, 72.7%), the number of participating institutions varied from 1 to 45 and included urban, rural, or mixed health care settings.

**Table 3.** Characteristics of study populations included in studies evaluating AI-based clinical decision support systems in primary care.

Authors	Context	Clinicians: sample size and characteristics	Patients (experimental group): sample size and characteristics	Patients (control group): sample size and characteristics	Number of participating institutions	Setting
Alcoceba-Herrero et al [76]	Acute phase of COVID-19	Clinician sample not described	n=170 Age:<30 y, n=13, 12.6%; 30‐45 y, n=39, 37.9%; 46‐65 y, n=46, 44.7%; >65 y, n=5, 4.9% Gender: n=51, 49.5% women	n=172 Age:<30 y, n=28, 19.4%; 30‐45 y, n=55, 38.2%; 46‐65 y, n=57, 39.6%; >65 y, n=4, 2.8% Gender: n=90, 62.5% women	25	Urban and rural
Areias et al [77]	Chronic musculoskeletal pain	Clinician sample not described	n=24,083 Age: mean 48.5 (SD 11.6) years Gender: women n=14,126, 58.7% Ethnicity: non-Hispanic White n=15,991, 67.9%	n=12,103 Age: mean 50.0 (SD 11.7) years Gender: women n=6984, 57.9% women Ethnicity: non-Hispanic White n=6013, 66.3%	Not reported	—^a^
Chen et al [78]	Shingles vaccination	Clinician sample not described	Not reported	Not reported	Not reported	Urban academic
Cruz et al [79]	Multiple conditions (hip or knee osteoarthritis, low back pain, headache, anxiety, osteoporosis, dyspepsia, heart failure, heart murmur, urinary tract infection)	n=86 primary care physicians and pediatricians	Not reported	Not reported	24	Not reported
Granviken et al [68]	Musculoskeletal pain	Experimental: n=22 physiotherapists Mean age 39.7 (SD 7.9) years Sex: 31.8% women Control: n=22 physiotherapists Mean age 40.8 (SD 8.8) years Sex: 27.3% women	n=358 Age: mean 47.0 (SD 14.9) years Sex: women n=238, 66.5%	n=366 Age: mean 49.8 (SD 15.1) years Sex: women n=226, 61.7%	33	Private
Herter et al [80]	Urinary tract infection	Clinician sample not described	Preimplementation: n=4998 Postimplementation: n=3422 Age:<30 y, 17%; 30‐50 y, 21%; 50‐70 y, 36%; >70 y, 26% Sex: 88% women Postimplementation: n=1121 with certainty of AI-CDSS use Age:<30 y, 15%; 30‐50 y, 18%; 50‐70 y, 39%; >70 y, 28% Sex: 92% women	Preimplementation: n=5044 Postimplementation: n=3360 Age:<30 y, 17%; 30‐50 y, 20%; 50‐70 y, 29%; >70 y, 33% Sex: 86% women	36 experimental; 29 control	Not reported
Liu et al [81]	Type 1 or type 2 diabetes mellitus	Clinician sample not described	n=180 Age: mean 57.4 (SD 11.4) years Gender: women n=97, 53.9% Ethnicity: Black n=141, 78.3%	n=974 Age: mean 56 (SD 13.8) years Gender: women n=565, 58% Ethnicity: Black n=746, 76.6%	1	Academic
Popescu et al [82]	Major depressive disorder	n=7 primary care physicians and psychiatrists	n=14 patients	Self-control	Not reported	—
Qassim et al [83]	Major depressive disorder	Not reported	Mean age 36.4 (SD 14.84) years Sex: women n=9, 64.3% Ethnicity: Caucasian n=10, 76.9 %	Not reported	Not reported	University hospitals, primary care clinics, and psychiatric clinics
Romero-Brufau et al [84]	Diabetes mellitus	Preimplementation: n=45 primary care physicians, advanced practice providers, nurses, clinical assistants, and others Postimplementation: n=38 primary care physicians, advanced practice providers, nurses, clinical assistants, and others	Not reported	Not reported	3	Not reported
Seol et al [85]	Asthma	n=42 residents and other primary care providers	n=90 Age: median 9.3 (Q1^b^=4.8; Q3^c^=13.3) years Sex: female, n=37, 41% Ethnicity: White n=68, 76%	n=94 Age: median 8.3 (Q1=5.4; Q3=12.7) years Sex: female, n=43, 46% Ethnicity: White n=64, 68%	1	Not reported
Rushlow et al [87]	Low ejection fraction	—	n=11,573	n=11,068	45	—
Thao et al [88]	Low ejection fraction	n=181 physicians, physician assistants, and nurse practitioners	Mean age 60.5 (SD 17.6) years	Mean age 60.5 (SD 17.5) years	—	Academic, community, and rural
Yao et al [86]	Low ejection fraction	Experimental: n=181 physicians, physician assistants, and nurse practitioners Age: mean 44.8 (SD 10.5) years Sex: 52.5% women Ethnicity: 94.4% White Control: n=177 physicians, physician assistants, and nurse practitioners Age: mean 46.1 (SD 11.01) years Sex: 59.7% women Ethnicity: 90.3% White	—	—	—	—

a Not applicable.

b Q1: first quartile.

c Q3: third quartile.

### Characteristics of the Interventions

We describe the control and experimental interventions in Table 4.

**Table 4.** Description of control and experimental interventions in studies evaluating AI-based clinical decision support systems in primary care.

Authors	Control intervention arm	Experimental intervention arm
Alcoceba-Herrero et al [76]	Clinical care: usual primary care follow-up for patients with COVID-19 in home isolation Delivery modality: scheduled telephone follow-up at 24 hours, 48 hours, 96 hours, 7 days, and 10 days; patient self-report of symptoms via a mobile app every 8 hours Decision support: none	Clinical care: same as control Delivery modality: same as control, with the addition of continuous remote monitoring of vital signs using wearable devices Decision support: automatic generation of alerts based on predefined thresholds; alerts triaged by severity (system-resolved, clinician, or emergency)
Areias et al [77]	Clinical care: digital Care Program for chronic MSK^a^ conditions Delivery modality: remote telerehabilitation platform; patient self-management with PT^b^ supervision Decision support: generation of recommendations for care adjustments; recommendation and rationale displayed to PT	Clinical care: same as control Delivery modality: same as control Decision support: none
Chen et al [78]	Clinical care: usual primary care practice with standard shingles vaccination alerts Delivery modality: alerts presented within the EHR^c^ interface Decision support: rule-based CDS^d^ alert system prompting shingles vaccination; all eligible alerts displayed to clinicians	Clinical care: same as control Delivery modality: same EHR interface and alert structure as control Decision support: prediction of the likelihood of clinician interaction with alerts; suppression of alerts predicted to be low-value based on a predefined threshold; alerts above the threshold displayed to clinicians
Cruz et al [79]	Clinical care: usual primary care practice Delivery modality: not reported Decision support: none	Clinical care: same as control Delivery modality: real-time tool used during consultation Decision support: processing of clinical notes from the EHR; generation of alerts with recommendations related to clinical pathways; alerts triggered when the patient case meets predefined criteria derived from clinical pathways translated into rules
Granviken et al [68]	Clinical care: usual physiotherapy care Delivery modality: in-person physiotherapy consultations Decision support: none	Clinical care: same as control Delivery modality: same as control, with the addition of a web-based CDSS^e^ used during consultation Decision support: identification of similar previous patients; generation of personalized treatment recommendations
Herter et al [80]	Clinical care: usual primary care practice Delivery modality: not reported Decision support: none	Clinical care: same as control Delivery modality: web-based decision support software used by general practitioners Decision support: patient characteristics entered manually into the system; estimation of expected outcomes for antibiotic treatment options based on similar patients; presentation of treatment options with predicted outcomes; display of guideline information alongside treatment options
Liu et al [81]	Clinical care: usual primary care practice Delivery modality: standard process (historical cohort from the same clinic) Decision support: none	Clinical care: same as control Delivery modality: fundus photography performed during the primary care visit Decision support: use of an automated retinal image analysis system; automated classification of diabetic retinopathy severity; referral recommendations based on results
Popescu et al [82]	Clinical care: usual primary care practice Delivery modality: not reported Decision support: none	Clinical care: same as control Delivery modality: use of a web-based clinical decision support system during clinical visits Decision support: patients complete questionnaires through the app; symptom tracking and visualization; provision of guideline-based treatment information at each visit; generation of individualized probabilities of remission for antidepressant treatments
Qassim et al [83]	Clinical care: usual primary care practice Delivery modality: not reported Decision support: none	Clinical care: same as control Delivery modality: use of a web-based clinical decision support system during clinical visits Decision support: patients complete questionnaires through the application; symptom tracking and visualization; provision of guideline-based treatment information at each visit; generation of individualized probabilities of remission for antidepressant treatments
Romero-Brufau et al [84]	Clinical care: usual primary care practice Delivery modality: standard workflow Decision support: none	Clinical care: same as control Delivery modality: use of an AI-based clinical decision support system implemented in primary care clinics Decision support: clinical assistants review lists of patients identified by the system before visits; patient risk profiles and recommendations are printed and provided to clinicians before the consultation; identification of patients at risk of poor glycemic control in the next 3 months; generation of recommendations intended to reduce risk
Seol et al [85]	Clinical care: usual primary care practice with or without enrollment in an Asthma Management Program Delivery modality: not reported Decision support: none	Clinical care: same as control Delivery modality: quarterly report provided to clinicians (every 3 mo) Decision support: prediction of 1-year risk of asthma exacerbation; provision of asthma management options to clinicians
Rushlow et al [87]	Clinical care: usual primary care practice Delivery modality: standard 12-lead ECG^f^ performed and interpreted as part of routine care Decision support: none	Clinical care: same as control Delivery modality: use of an AI algorithm that was automatically applied to ECGs performed during routine care Decision support: clinicians received alerts when patients had a positive AI-ECG^g^ result; ECGs were classified as positive or negative for low ejection fraction; clinicians were advised to consider ordering an echocardiogram for positive results; recommendations were provided within the report; weekly email reminders were sent when no action was taken following a positive result
Thao et al [88]	Clinical care: usual primary care practice Delivery modality: standard 12-lead ECG performed and interpreted as part of routine care Decision support: none	Clinical care: same as control Delivery modality: use of an AI algorithm that was automatically applied to ECGs performed during routine care Decision support: clinicians received alerts when patients had a positive AI-ECG result; ECGs were classified as positive or negative for low ejection fraction; clinicians were advised to consider ordering an echocardiogram for positive results; recommendations were provided within the report; weekly email reminders were sent when no action was taken following a positive result
Yao et al [86]	Clinical care: usual primary care practice Delivery modality: standard 12-lead ECG performed and interpreted as part of routine care Decision support: none	Clinical care: same as control Delivery modality: use of an AI algorithm that was automatically applied to ECGs performed during routine care Decision support: clinicians received alerts when patients had a positive AI-ECG result; ECGs were classified as positive or negative for low ejection fraction; clinicians were advised to consider ordering an echocardiogram for positive results; recommendations were provided within the report; weekly email reminders were sent when no action was taken following a positive result

a MSK: musculoskeletal.

b PT: physiotherapist.

c EHR: electronic health record.

d CDS: clinical decision support.

e CDSS: clinical decision support system.

f ECG: electrocardiogram.

g AI-ECG: AI-enhanced electrocardiogram.

Of the 11 studies, control interventions were mainly usual primary care practice (n=10, 90.9%). The remaining study had a digital care program as the control group. One study used a rule-based CDSS as the control intervention arm. Experimental interventions generally preserved the same clinical care as the control while adding an AI-CDSS.

We present the identified AI-CDSS in Table 5.

**Table 5.** Overview of AI-based clinical decision support systems (AI-CDSSs) evaluated in primary care, including system names, intended purpose, and training or support provided for their use.

Authors	Name of the AI-CDSS	Purpose	Training or support to use AI-CDSS
Alcoceba-Herrero et al [76]	No name	Support early detection of patient deterioration in COVID-19 home isolation	Technical, logistical, and clinical support
Areias et al [77]	No name	Support monitoring and adjustment of digital care programs for individuals living with chronic musculoskeletal pain	Not reported
Chen et al [78]	SmartCDS^a^	Reduce alert fatigue and increase the signal-to-noise ratio of shingles vaccination reminders	Not reported
Cruz et al [79]	No name	Support guideline adherence for multiple conditions	Not reported
Granviken et al [68]	SupportPrim PT^b^ CDSS^c^	Support personalized decision-making for musculoskeletal pain management	Training
Herter et al [80]	No name	Support selection of the most effective antibiotic for urinary tract infections	Training
Liu et al [81]	EyeArt v2.0 ARIAS^d^	Support early detection of diabetic retinopathy and improve adherence to follow-up eye care	No
Popescu et al [82]	Aifred	Support treatment selection and management for major depressive disorders	Training
Qassim et al [83]	Aifred	Support treatment selection and management for major depressive disorders	Training
Romero-Brufau et al [84]	No name	Support glycemic control in patients with diabetes	Not reported
Seol et al [85]	No name	Support asthma management in children	Not reported
Rushlow et al [87]	No name	Support early detection of low ejection fraction in adults without prior heart failure	Website access with videos and FAQs^e^
Thao et al [88]	No name	Support early detection of low ejection fraction in adults without prior heart failure	Website access with videos and FAQs^e^
Yao et al [86]	No name	Support early detection of low ejection fraction in adults without prior heart failure	Website access with videos and FAQs^e^

a CDS: clinical decision support.

b PT: physiotherapist.

c CDSS: clinical decision support system.

d ARIAS: Automated Retinal Image Analysis System.

e FAQ*:* frequently asked question.

Studies evaluated 11 distinct AI-CDSSs with different purposes. Seven (63.6%) AI-CDSSs aimed to support condition management, 3 (27.3%) aimed at the early detection of a condition or a deterioration, and 1 (9.1%) aimed to reduce alert fatigue. Preimplementation training occurred in 3 (27.3%) studies, and implementation support occurred in 2 (18.2%) studies.

We provide AI characteristics of the identified AI-CDSS in Table 6.

**Table 6.** AI characteristics of AI-based clinical decision support systems evaluated in primary care, including model accessibility, AI system type, year of model development, and interoperability with clinical systems.

Authors	AI model accessibility	AI system type	AI development date	Interoperability
Alcoceba-Herrero et al [76]	Not reported	Not reported	Not reported	Not reported
Areias et al [77]	Commercial	Machine learning	Not reported	Not reported
Chen et al [78]	Institutional but not open source	Machine learning	Not reported	EHR^a^ embedded
Cruz et al [79]	Commercial	Natural language processing and rule-based system	Not reported	Uses EHR data and integration within EHR interface not reported
Granviken et al [68]	Not reported	Case-based reasoning	Not reported	Stand-alone system
Herter et al [80]	Commercial	Machine learning	Not reported	Uses EHR data and integration within EHR interface not reported
Liu et al [81]	Commercial	Deep learning^b^	Not reported	Stand-alone system
Popescu et al [82]	Commercial	Deep learning	Not reported	Stand-alone system
Qassim et al [83]	Commercial	Deep learning	Not reported	Stand-alone system
Romero-Brufau et al [84]	Commercial	Not reported	Not reported	Not reported
Seol et al [85]	Not reported	Machine learning	Not reported	Uses EHR data and outputs delivered via external reports (integration within EHR interface not reported)
Rushlow et al [87]	Institutional but not open source	Deep learning	Not reported	EHR embedded
Thao et al [88]	Institutional but not open source	Deep learning	Not reported	EHR embedded
Yao et al [86]	Institutional but not open source	Deep learning	Not reported	EHR embedded

a EHR: electronic health record.

b Assumed based on study description.

Six (54.5%) identified AI-CDSSs were commercial products, 2 (18.2%) were institutionally developed but not open source, and 3 (27.3%) did not report the accessibility of the underlying AI method. Nine (81.8%) studies reported the type of AI system; among these, 4 (44.4%) used machine learning, and 3 (33.3%) used deep learning approaches. No study reported when the AI model was developed. Regarding interoperability, 4 (36.4%) studies evaluated stand-alone systems, 2 (18.2%) reported electronic health record (EHR)–embedded systems, and 3 (27.3%) used EHR data without specifying integration within the EHR interface. Three studies (27.3%) did not report interoperability information.

We present the CDSS characteristics in Table 7.

**Table 7.** Functional and operational characteristics of AI-based clinical decision support systems evaluated in primary care, including clinical decision support system (CDSS) functions, operational functions, mode of delivery, additional tasks required, and final decision-maker.

Authors	CDSS functions	Operational functions	Mode of delivery	Additional tasks	Final decision made by
Alcoceba-Herrero et al [76]	Point-of-care alerts or reminders Relevant information display	Alert optimization Documentation or care summary Monitoring or follow-up	Dashboard Alerts	Review dashboard	Clinicians^a^
Areias et al [77]	Expert systems Relevant information display	Documentation/care summary Monitoring/follow-up Treatment optimization	Dashboard	Review and navigate dashboard	Clinicians
Chen et al [78]	Point-of-care alerts/reminders	Alert optimization	Alerts	No	N/A^b^
Cruz et al [79]	Point-of-care alerts/reminders	Treatment optimization	Alerts	No	Clinicians^a^
Granviken et al [68]	Expert systems Relevant information display	Treatment optimization	Dashboard	Review and navigate dashboard	Clinicians^a^
Herter et al [80]	Expert systems Relevant information display	Risk prediction Treatment optimization	Dashboard	Enter patient’s data Review bar chart	Clinicians^a^
Liu et al [81]	Expert systems	Referral Screening	No information	Capture fundus images^a^	Clinicians^a^
Popescu et al [82]	Expert systems	Documentation/care summary	Dashboard	Review and navigate dashboard	Clinicians
Qassim et al [83]	Relevant information display	Monitoring/follow-up Risk prediction Treatment optimization	Dashboard	Review and navigate dashboard	Clinicians
Romero-Brufau et al [84]	Expert systems Relevant information display	Risk prediction Screening Treatment optimization	Printed report	Clinical assistant print the report Review the report	Clinicians
Seol et al [85]	Expert systems Relevant information display	Documentation/care summary Monitoring/follow-up Risk prediction Treatment optimization	Quarterly report	No	Clinicians^a^
Rushlow et al [87]	Expert systems Point-of-care alerts/reminders Relevant information display	Referral Risk prediction Screening	Email alerts	No	Clinicians^a^
Thao et al [88]	Expert systems Point-of-care alerts/reminders Relevant information display	Referral Risk prediction Screening	Email alerts	No	Clinicians^a^
Yao et al [86]	Expert systems Point-of-care alerts/reminders Relevant information display	Referral Risk prediction Screening	Email alerts	No	Clinicians^a^

a Assumed based on study description.

b N/A: not applicable.

Expert system functions and relevant information display were each reported in 8 (72.7%) studies, and point-of-care alerts or reminders in 4 (36.4%). Eight (72.7%) of the 11 AI-CDSSs included multiple functions. These functions translated into operational capabilities, such as treatment optimization (7/11, 63.6%), risk prediction (5/11, 45.5%), monitoring or follow-up (4/11, 36.4%), documentation or care summary (4/11, 36.4%), screening (3/11, 27.3%), referral (2/11, 18.2%), and alert optimization (2/11, 18.2%). Modes of delivery included dashboards (5/11, 45.5%), alert-based systems (3/11, 27.3%), printed or periodic reports (2/11, 18.2%), and email notifications (1/11, 9.1%). One (9.1%) study did not report the mode of delivery. All systems aimed to support rather than replace clinician decision-making, as explicitly stated in 3 (27.3%) studies and implicit in the remainder. In 7 (63.6%) studies, AI-CDSS use required additional clinician tasks, including access to and review of dashboards (5/11, 45.5%), manual data entry (1/11, 9.1%), acquisition of clinical images (1/11, 9.1%), printing of reports (1/11, 9.1%), and review of system-generated reports (1/11, 9.1%).

Using the classification approach adapted from the NASSS framework, included studies were assigned to simple (4/11, 36.4%), complicated (4/11, 36.4%), and complex (3/11, 27.2%) implementation contexts (see Table 8 and Multimedia Appendix 3 for rating details).

**Table 8.** Classification of implementation contexts for AI-based clinical decision support systems in primary care using the simple–complicated–complex typology informed by the NASSS^a^ framework.

Authors	Classification	Justification
Alcoceba-Herrero et al [76]	Simple	Condition: clinically defined acute infectious condition (COVID-19) with relatively standardized monitoring targets Decision logic: protocolized (predefined thresholds for alerts and escalation) System: operationally simple (remote monitoring+app+automated alerts, but rule-based and predictable) Preference sensitivity: low (decisions driven by clinical deterioration thresholds rather than patient preferences)
Areias et al [77]	Complex	Condition: clinically heterogeneous chronic MSK^b^ conditions with variable presentations and trajectories Decision logic: individualized (continuous adjustment of care based on patient response and clinician input) System: multicomponent adaptive system (AI support+digital care program+PT^c^ supervision+longitudinal monitoring) Preference sensitivity: high (treatment adaptation depends on patient goals, engagement, and response)
Chen et al [78]	Simple	Condition: narrow preventive care task (shingles vaccination eligibility) Decision logic: protocolized (rule-based alert suppression optimizing signal-to-noise) System: operationally simple (EHR^d^-integrated alert system) Preference sensitivity: low (clear guideline-based action)
Cruz et al [79]	Complicated	Condition: multiple conditions with varying levels of clinical heterogeneity Decision logic: protocolized (guideline-based pathway adherence recommendations) System: integrated structured system (NLP^e^ processes EHR data and generates real-time recommendations, but not fully embedded within the EHR workflow interface) Preference sensitivity: low to high depending on the conditions
Granviken et al [68]	Complex	Condition: clinically heterogeneous MSK pain with variable prognosis and response Decision logic: individualized (treatment decisions require integration of multiple patient factors) System: multicomponent adaptive system (case-based reasoning+clinician judgment+patient variability) Preference sensitivity: high (explicit emphasis on shared decision-making and person-centered care)
Herter et al [80]	Complicated	Condition: moderately heterogeneous condition (UTI^f^ presentations and risk profiles) Decision logic: structured (prediction-based guidance using decision trees within bounded decision space) System: integrated structured system (uses EHR data, but not described as fully embedded within the EHR workflow interface) Preference sensitivity: low to moderate (some trade-offs, but guideline constrained)
Liu et al [81]	Simple	Condition: well-defined screening target (diabetic retinopathy) Decision logic: protocolized (binary classification → referral vs no referral) System: operationally simple (point-of-care screening+automated classification) Preference sensitivity: low (screening/referral decision is guideline driven)
Popescu et al [82]	Complex	Condition: clinically heterogeneous mental health condition Decision logic: structured+individualized (guideline-based algorithm combined with personalized AI predictions) System: multicomponent adaptive system (AI predictions+measurement-based care+clinician interpretation) Preference sensitivity: high (treatment selection depends on patient preferences, tolerance, and context)
Qassim et al [83]	Complex	Condition: clinically heterogeneous mental health condition Decision logic: structured+individualized (guideline-based algorithm combined with personalized AI predictions) System: multicomponent adaptive system (AI predictions+measurement-based care+clinician interpretation) Preference sensitivity: high (treatment selection depends on patient preferences, tolerance, and context)
Romero-Brufau et al [84]	Complicated	Condition: moderately heterogeneous chronic condition (diabetes with varying control levels) Decision logic: structured (risk stratification+recommended interventions) System: integrated structured system (AI identifies high-risk patients and suggests actions) Preference sensitivity: moderate (some contextual tailoring needed but within structured care pathways)
Seol et al [85]	Complicated	Condition: clinically heterogeneous but stratified (childhood asthma with severity-based grouping) Decision logic: structured (risk prediction+periodic report guiding management decisions) System: integrated structured system (quarterly reports+ML^g^ predictions within workflow) Preference sensitivity: low to moderate (decisions guided by risk but still clinician mediated)
Rushlow et al [87]	Simple	Condition: well-defined diagnostic target (low ejection fraction) Decision logic: protocolized (AI screening → echocardiography referral) System: operationally simple (screening tool embedded in routine ECG^h^ workflow) Preference sensitivity: low (diagnostic confirmation pathway is standardized)
Thao et al [88]	Simple	Condition: well-defined diagnostic target (low ejection fraction) Decision logic: protocolized (AI screening → echocardiography referral) System: operationally simple (screening tool embedded in routine ECG workflow) Preference sensitivity: low (diagnostic confirmation pathway is standardized)
Yao et al [86]	Simple	Condition: well-defined diagnostic target (low ejection fraction) Decision logic: protocolized (AI screening → echocardiography referral) System: operationally simple (screening tool embedded in routine ECG workflow) Preference sensitivity: low (diagnostic confirmation pathway is standardized)

a NASSS: Non-Adoption, Abandonment, Scale-Up, Spread, and Sustainability.

b MSK: musculoskeletal.

c PT: physiotherapist.

d EHR: electronic health record.

e NLP: natural language processing.

f UTI: urinary tract infection.

g ML: machine learning.

h ECG: electrocardiogram.

### Outcomes and Effects

#### Overview

We present a summary of between-group effects in Table 9 and provide a detailed table, including within-group results, in Multimedia Appendix 8.

Among the 11 included studies, outcomes were most frequently reported at the clinician level (n=8, 72.7%), followed by the system level (n=6, 54.5%) and the patient level (n=5, 45.4%). Nearly half (n=5, 45.4%) of the studies assessed outcomes across all 3 levels.

**Table 9.** Between-group effects of AI-based clinical decision support systems on clinician-, patient-, and system-level outcomes in primary care, by study design and implementation context.

Study	Implementation context	Clinician-level effects	Patient-level effects	System-level effects
Experimental studies				
Alcoceba-Herrero et al [76]	Simple	During implementation period (~2 mo): reduction of alerts^a^	—^b^	—
Granviken et al [68]	Complex	During implementation period (~9 mo): more treatment modalities^a^	At week 12: global perceived effect^c^; pain intensity^c^; pain self-efficacy^c^; emotional distress^c^; general MSK^d^ health^c^; number of pain sites^c^; health-related quality of life^c^; sleep^c^; vitality^c^; use of pain medication in the last week^c^; functional status^e^; work ability^e^	During implementation period (~9 mo): number of consultations^c^
Seol et al [85]	Complicated	During implementation period (~12 mo): reduction of clinician burden^a^	At 12 mo: occurrence of asthma^c^ Assessed at 4 time points during the implementation period: asthma control status^c^	During implementation period (~12 mo): health care costs^c^; timeliness of follow-up after exacerbation^c^
Yao et al [86], Rushlow et al [87], and Thao et al [88]	Yao et al [86] and Rushlow et al [87]: —; Thao et al [88]: simple	—	During implementation period (~8 mo): newly diagnosed low ejection fraction^a^	During implementation period (~8 mo): echocardiography utilization for high-risk patients^a^; cost-effectiveness^a^; echocardiography utilization for overall cohort^c^
Quasi-experimental studies				
Areias et al [77]	Complex	—	At week 12: satisfaction with the program^a^; pain response rate^c^; mental health^c^; pain intensity^e^	During implementation period (~6 mo): completion rate of the program^a^; reach outs^a^; number of messages initiated by patients^a^; PT^f^-to-patient ratios^a^; safety^c^; sessions performed per week^e^
Chen et al [78]	Simple	During implementation period (~2 mo): order count^c^; interacted alert counts^c^	—	—
Cruz et al [79]	Complicated	During implementation period (~1 mo): adherence rate to 3 recommendations^a^; adherence rate to 5 recommendations^c^	—	—
Herter et al [80]	Complicated	During implementation period (~4 mo): prescription behavior^a^^,^^c^^,^^e^	During implementation period (~4 mo): successful treatment^a^	—
Liu et al [81]	Simple	—	—	During implementation period (~8 mo): rate of attendance at ophthalmology appointments^a^
Popescu et al [82] and Qassim et al [83]	Popescu et al [82]: —; Qassim et al [83]: complex	During implementation period (~10 mo): appointment length^c^	—	—
Romero-Brufau et al [84]	Complicated	At the end of implementation period (~1 mo): Clinician attitudes: care coordination^a^; patient preparation for managing diabetes^a^; familiarity with AI^a^; reducing complications^c^; intervention effectiveness^c^; right intervention^c^; routinely use AI^c^; AI will complicate my job^c^; AI will make my job obsolete^c^; excited about AI^e^; AI does not understand my job^e^	—	—

a Favors the experimental group.

b Not applicable.

c No difference.

d MSK: musculoskeletal*.*

e Favors the control group.

f PT: physiotherapist.

#### Clinician-Level Outcomes

Four (50%) studies reported workflow outcomes assessed during the implementation period, resulting in mixed effects. Two experimental studies reported reductions in alert burden and clinician workload across implementation periods ranging from short (~2 mo) to longer periods (~12 mo). These studies were conducted using protocolized or structured decision logic. Two quasi-experimental studies reported no effect on interacted alert counts in a simple context with an automated EHR-embedded AI-CDSS for alert optimization, and no effect on appointment length in a complex context characterized by individualized decision logic, a multicomponent system, and high preference sensitivity.

Four (50%) studies reported decision behavior outcomes during the implementation period. One experimental and 3 quasi-experimental studies examined prescribing behavior, guideline adherence, or order count with heterogeneous effects across and within studies. These studies were conducted across different implementation contexts. In the simple context, alert suppression was not associated with changes in the order count outcome. Two studies conducted in complicated contexts reported mixed effects on prescribing behavior and positive or neutral effects on adherence to clinical guidelines. The experimental study conducted in a complex context reported increased use of treatment modalities. These complicated and complex contexts were characterized by clinical heterogeneity, structured or individualized decision logic, and moderate to high preference sensitivity.

One quasi-experimental study (n=1, 12.5%) context evaluated attitudinal outcomes at the end of the short implementation period (~1 mo) in a complicated context, reporting improvements in care coordination and patient preparation, alongside concerns regarding the usefulness of the system and its impact on clinical work.

#### Patient-Level Outcomes

Two (40%) studies reported patient-reported outcomes assessed at 12 weeks in musculoskeletal conditions. Pain intensity and mental health were the only outcomes assessed in more than 1 study. Across these studies, findings for pain intensity were inconsistent, with 1 experimental study reporting no effect and 1 quasi-experimental study reporting a negative effect. Both studies reported no effect on mental health outcomes. These studies were conducted in complex contexts characterized by clinical heterogeneity, individualized decision logic, multicomponent systems, and high preference sensitivity.

Two (40%) studies reported clinical outcomes assessed during or after the study period. One experimental study reported no effect on asthma occurrence and control, while 1 quasi-experimental study observed a positive effect on treatment success. Both studies were conducted in complicated contexts characterized by clinically heterogeneous but stratified conditions, protocolized decision logic, and moderate preference sensitivity, and differed in system complexity.

Two (40%) studies reported process-of-care outcomes with mixed effects. One experimental study reported a positive effect on the detection rate of low ejection fraction in a simple implementation context characterized by a well-defined diagnostic target, protocolized decision logic, an operationally simple system, and low preference sensitivity. An experimental study reported no effect on medication use in a complex context involving clinical heterogeneity, individualized decision logic, a multicomponent system, and high preference sensitivity.

One quasi-experimental study (1/5, 20%) reported a patient-reported experience outcome (satisfaction with the program) at 12 weeks with a positive effect.

#### System-Level Outcomes

Three (50%) studies evaluated service delivery outcomes during the implementation period and reported mixed results. One quasi-experimental study reported improvements in program completion and patient engagement (eg, reach-outs and messages), while another quasi-experimental study found increased attendance at follow-up appointments. These effects were observed across different implementation contexts, including both complex and simple contexts. Two studies reported neutral or negative effects on service delivery indicators, such as the number of consultations (experimental study) and sessions performed per week (quasi-experimental study), both conducted in complex contexts.

Two (33.3%) experimental studies evaluated economic outcomes with mixed effects. One study reported that the intervention was cost-effective, while the other found no effect on health care costs. The 2 studies were conducted in different implementation contexts, including 1 simple context involving a well-defined diagnostic target with low preference sensitivity and 1 complicated context with a heterogeneous condition and moderate preference sensitivity.

Two (33.3%) studies reported utilization outcomes with positive effects. An experimental study reported an increased use of diagnostic procedures among high-risk patients but no clinical effect difference in the intervention sample. This result was observed in a simple context characterized by a well-defined diagnostic target and a protocolized decision logic. One quasi-experimental study reported improved physiotherapist-to-patient ratios in a complex context.

One (16.7%) quasi-experimental study reported no impact on safety during the implementation period in a complex context.

### Safety Evaluation

We present the reporting of safety-related information in Table 10. Two (18.2%) studies prespecified structured monitoring of adverse events, primarily relying on patient-reported side effects or standardized questionnaires rather than active surveillance of AI-related harms. Across these 2 studies, as well as 1 additional study reporting post hoc harms and unintended effects, no severe adverse events attributable to the AI-CDSS were identified. Risk mitigation strategies were described in 4 (36.4%) studies and included human-in-the-loop oversight, internal quality checks, and automated safeguards such as validation of severe alerts or continuous retraining with monitoring dashboards. Reports of errors or malfunctions were uncommon (n=3, 27.3%) and mainly incidental, involving image quality issues, software glitches, or technical exclusions of participants. No study reported clinician agreement with or override of AI-generated recommendations.

**Table 10.** Reporting of safety-related information for AI-based clinical decision support systems in primary care at patient level, system level, and clinician level.

Authors	Patient safety: prespecification	Patient safety: harms and unintended effects	AI system: risk mitigation	AI system: significant errors and malfunctions	Clinician interaction: agreement and override
Alcoceba-Herrero et al [76]	Not reported	Not reported	Patient validation required before escalation of severe alerts to emergency	Not reported	Not reported
Areias et al [77]	Report of any adverse events	Intervention-related adverse events were rare and were evenly distributed across the groups	Human-in-the-loop Routine internal quality checks comparing algorithms versus clinicians’ decisions	Not reported	Not reported
Chen et al [78]	Not reported	Not reported	Monitoring dashboard for alert outputs Model retrained daily	Not reported	Not reported
Cruz et al [79]	Not reported	Not reported	Not reported	Not reported	Not reported
Granviken et al [68]	Not reported	Not reported	Not reported	Two participants in the intervention group excluded due to technical issues	Not reported
Herter et al [80]	Not reported	Not reported	Not reported	Not reported	Not reported
Liu et al [81]	Not reported	Not reported	Not reported	Errors related to image quality reported (eg, ungradable/poor-quality images)	Not reported
Popescu et al [82]	Weekly collection of PRISE-20^a^ and FIBSER^b^ to assess side effects and UKU^c^ scale after each visit	No serious adverse events related to the tool	Software errors identified during the trial were corrected	Incorrect questionnaire score comparison in CANMAT^d^ algorithm	Not reported
Qassim et al [83]	Weekly collection of PRISE-20 and FIBSER to assess side effects and UKU scale after each visit	No serious adverse events related to the tool	Software errors identified during the trial were corrected	Broken link between pages in the CANMAT algorithm	Not reported
Romero-Brufau et al [84]	Not reported	Not reported	Not reported	Not reported	Not reported
Seol et al [85]	Not reported	0 serious adverse event	Not reported	Not reported	Not reported
Yao et al [86]	Not reported	Not reported	Not reported	Not reported	Not reported
Rushlow et al [87]	Not reported	Not reported	Not reported	Not reported	Not reported
Thao et al [88]	Not reported	Not reported	Not reported	Not reported	Not reported

a PRISE-20: Patient-Rated Inventory of Side Effects-20 items.

b FIBSER: Frequency, Intensity, and Burden of Side Effects Rating scale.

c UKU: Udvalg for Kliniske Undersøgelser.

d CANMAT: Canadian Network for Mood and Anxiety Treatments.

## Discussion

### Principal Findings

This systematic review synthesized evidence on the effectiveness and safety of AI-CDSSs in primary care across 11 studies. Two RCTs, conducted in a simple implementation context with screening or alert optimization AI-CDSSs, reported positive effects on workflow, economic, and utilization outcomes. In contrast, studies conducted in more complex contexts reported limited or inconsistent effects, with patient-level outcomes rarely improved. Across studies, most systems were deployed as stand-alone tools requiring additional clinician effort, and follow-up periods were generally short, reflecting early phases of implementation. Safety was not evaluated as an integrated, multilevel construct. Only 2 studies prespecified patient safety outcomes. System-level safeguards were described but not evaluated, and no study examined how clinicians engaged with AI-generated recommendations. The current evidence base does not clarify how system performance, clinician behavior, and patient outcomes interact to shape safety in practice. The overall strength of evidence is further limited by substantial heterogeneity and consistently high or critical risk of bias, restricting confidence in the observed effects and their applicability across clinical contexts.

### Effectiveness Considerations

Positive effects of AI-CDSS implementation were reported more consistently when clinical decisions followed structured and protocolized action pathways. In our review, the most consistent effects emerged in studies targeting screening, referral, or alert optimization, where clinical rules were explicit and thresholds were predefined. In these contexts, recommendations could be readily integrated into routine workflows and translated into immediate clinical responses. Prior CDSS research similarly suggests that decision support is more likely to improve care when recommendations are delivered at the point of care, embedded within clinical workflows, and linked to clear actions [89-91]. However, structured decision contexts alone may be insufficient to ensure effectiveness, as systems that increase workflow burden, generate excessive interruptions, or poorly align with local practices may reduce clinician responsiveness over time [92-96]. Alert fatigue represents a well-documented failure mode, with studies reporting override rates exceeding 50% to 73% for medication-related alerts, with clinicians becoming progressively less likely to accept alerts as their frequency increases [94,97,98]. This suggests that the effects of AI-CDSSs in structured contexts may depend not only on the clarity of the underlying clinical rules but also on the degree to which system recommendations remain actionable, relevant, and aligned with the realities of clinical practice.

In settings requiring complex or individualized decision-making, AI-CDSS effects on clinical outcomes appear less consistent, potentially not because the systems fail, but because they are used differently in ways that leave a limited measurable trace. Clinicians integrating evolving clinical information, patient goals, and preference-sensitive trade-offs across longitudinal care trajectories face decisions that are less amenable to reduction into a single rule, threshold, or predefined action pathway [99-101]. Several mechanisms may contribute to this pattern. First, as decision tasks become more cognitively demanding, clinicians appear less likely to consult decision support systems and may shift toward situation-assessment tools rather than algorithmic recommendations [102]. Second, physicians tend to remain anchored to pre-existing diagnoses when using diagnostic CDSSs. Berner et al [103] found that system outputs more often reinforced than redirected clinical reasoning, a pattern consistent with confirmation and anchoring biases previously described in clinical reasoning research, particularly under cognitively demanding conditions [104,105]. Third, even when systems are consulted, clinicians may resist outputs perceived as undermining their decision autonomy or conflicting with the clinical context, particularly when system reasoning is opaque or inconsistent [106,107]. These mechanisms may shift AI-CDSSs from a directive toward a more interpretive or confirmatory role. In our review, a study targeting chronic musculoskeletal pain found that clinicians used AI-CDSSs primarily to support existing practice rather than to modify management decisions [68]. This pattern is consistent with broader evidence showing that CDSS effects on clinical outcomes in chronic disease are often modest and heterogeneous even when improvements in process measures are observed [108,109]. If AI-CDSS outputs primarily validate or refine reasoning rather than consistently triggering management changes, measurable downstream effects on clinical outcomes may be expected to remain small, variable, and difficult to detect. Decision complexity should be explicitly addressed during AI-CDSS design, implementation, and evaluation [29]. Future research may also need evaluation approaches that better capture interpretive and confirmatory influences on clinical reasoning rather than relying exclusively on downstream outcome measures.

Implementation success also depends on alignment with organizational context, clinical workflows, and clinician routines [7,53,110]. Many AI-CDSS failures appear to be sociotechnical rather than algorithmic [19,29,111-113]. In our review, most AI-CDSSs were implemented as stand-alone systems requiring additional clinician effort, including manual data entry, dashboard consultation, or review of system-generated outputs outside existing workflows. In primary care, increased workload consistently emerges as a major barrier to CDSS adoption, with human and organizational factors negatively influencing uptake regardless of technological performance [7]. Workflow integration, usability, and health care system–level factors have also been identified as persistent implementation challenges across clinical domains and CDSS types [53,110], reinforcing the importance of aligning decision support systems with routine clinical work [29,32]. These barriers may become particularly consequential in more complex care settings, where workflows are often less standardized. In these contexts, clinicians must continuously integrate evolving clinical and contextual information across encounters, providers, and competing patient priorities, as in chronic disease management, multimorbidity, or preference-sensitive care [52]. EHR integration is frequently proposed as a strategy to reduce these implementation barriers [111,113], and limited evidence from our review is consistent with this interpretation, as the 2 interventions described as EHR-integrated both reported positive outcomes. However, the small number of studies prevents causal interpretation, and integration alone may be insufficient to ensure effectiveness. A CDSS deployment study reported implementation failure despite high algorithmic performance because only a minority of required input variables were available at the point of care, limiting the system’s practical usability during clinical decision-making [114]. EHR integration may also constrain decision support functionality or require substantial local infrastructure investment, particularly in fragmented primary care systems [112,113].

Interpretation of these findings should also consider systematic differences in outcome selection and evaluation timing across implementation contexts. In our review, AI-CDSSs implemented in structured contexts primarily evaluated process-level outcomes, whereas patient-level clinical outcomes were less frequently assessed, a pattern consistent with prior reviews [115,116]. Process outcomes are more readily measurable within short evaluation windows and are often more immediately responsive to decision support interventions [89,116]. However, improvements in process-level outcomes do not necessarily translate into patient-relevant benefit [89,116,117]. Reliance on intermediate end points has been associated with overestimated effects compared with trials evaluating patient outcomes directly [118]. In individualized contexts, where outcomes are shaped by interacting clinical and contextual factors across longitudinal care trajectories, isolating change specifically to AI-CDSSs becomes methodologically challenging [119]. Short evaluation timing was common in our review and may have captured early implementation phases before stable adoption patterns were established. Clinician engagement with decision support tends to evolve over time as familiarity, workflow demands, and implementation barriers change [31,120,121]. These interpretive challenges may partly reflect a mismatch between the predominantly point-in-time logic of current AI-CDSSs and the longitudinal structure of primary care. Most systems in our review were designed to support discrete decisions. This approach is well suited to structured tasks but less aligned with care that unfolds across encounters, depends on evolving patient goals, and requires ongoing adaptation to changing clinical circumstances [122,123]. Achieving more consistent effects in complex primary care settings may require forms of decision support evolving along with care trajectories, clinician judgment, and shared decision-making. Evaluation strategies may also need longer follow-up periods and clearer prespecified linkage between process-level changes and patient-level outcomes.

### Safety Considerations

Current reporting of AI-CDSS implementation research captures safety through fragmented and inconsistently specified indicators. Only 2 studies in this review prespecified safety outcomes, and most relied on the absence of serious adverse events as a proxy for safety, with limited detail regarding how harms were identified, monitored, or attributed. This approach risks missing harms that are subtle, cumulative, or delayed, such as missed follow-up, delayed diagnosis, or inappropriate treatment, because these events often require prospective monitoring rather than retrospective absence of signals [124]. In primary care, these risks are amplified by the longitudinal and distributed nature of care [125]. Clinical presentations are heterogeneous, care unfolds across repeated encounters, and adverse outcomes often cannot be attributed to a single decision or clinician interaction [126,127]. Diagnostic delays in ambulatory care, for example, frequently emerge from failures in follow-up coordination and clinical sensemaking across complex work systems rather than from isolated technical errors [128]. Similar concerns have been raised across the broader AI-CDSS literature, where safety reporting often remains secondary to performance evaluation, and benefits and harms are frequently assessed separately, limiting interpretation of overall clinical impact [34,129,130]. Recent expert consensus statements have also highlighted the absence of structured adverse event reporting methodologies and postdeployment monitoring strategies for AI systems in clinical care [38], while calls for systematic “algorithmovigilance” remain only partially operationalized in practice [37].

Our findings further suggest that AI-CDSS safety in primary care cannot be adequately understood through isolated technical or patient-level indicators. Safety events involving digital systems characteristically emerge from interactions among technical performance, clinician behavior, workflow conditions, and organizational context [93,126,131,132]. These dimensions of safety were largely unreported in the studies included in our review. This gap may be particularly important in primary care, where high cognitive load and cumulative alert exposure increase the likelihood of automation bias and alert fatigue [94,133,134]. Harm in primary care can also often accumulate longitudinally across encounters rather than concentrating in discrete events detectable through standard adverse event monitoring [125,128]. Without structured and adapted safety evaluation, implementation studies risk producing evidence for AI-CDSS adoption without establishing whether these systems are, in practice, safe to use. These features of primary care point to evaluation requirements that are not generic but structurally specific to the setting. These include prospective specification of safety outcomes inclusive of process-level harms such as missed follow-up and delayed referral [38,65]; longitudinal monitoring during routine use rather than at deployment alone [127,135]; systematic reporting of clinician engagement with AI recommendations, including override patterns [94,133]; and monitoring intensity calibrated to the clinical consequence of supported decisions, which in primary care spans chronic disease management to acute triage [65,136]. Without evaluation approaches adapted to these features of primary care, the growing body of AI-CDSS implementation evidence risks establishing effectiveness without establishing safety, reversing the expected evidence sequence in clinical research [55].

This review has subject-matter and methodological limitations. First, our search strategy was developed in collaboration with an information specialist (FB) and tested for sensitivity. However, we identified several studies through citation searching and trial registration number. This reflects the indexing inconsistencies and incomplete database coverage for CDSS, despite the existence of a MeSH term since 1997. Our methodology, including backward and forward citation screening in identified knowledge syntheses records, reduces the risk of missing studies that would affect the overall result interpretation. As AI-CDSS implementation studies continue to emerge, the evidence synthesized in this review reflects the literature available up to the predefined search dates (June 27, 2025). Future work should periodically repeat this search approach to monitor newly published evaluations and incorporate emerging evidence over time. Second, substantial heterogeneity across interventions, study designs, implementation contexts, and reported outcomes precluded meta-analysis and limited the use of summary effect estimates for strength-of-evidence interpretation. We therefore used synthesis without meta-analysis based on direction of effect. As emphasized in the SWiM guidance [71], this synthesis approach answers whether there is evidence of effects across studies, rather than estimating an average intervention effect. Our findings should be interpreted as patterns of reported effects across heterogeneous AI-CDSS implementations, not as a pooled estimate of AI-CDSS effectiveness in primary care. Although this approach was appropriate given the available evidence, it carries a risk of interpretive bias. We mitigated this risk through structured outcome grouping, predefined implementation context criteria, and review by a multidisciplinary team of researchers, clinicians, and people with lived experience. Beyond these methodological considerations, interpretation of the findings should also consider the well-recognized international variation in the organization and delivery of primary care across health systems [1,137]. Although all included studies met our predefined eligibility criteria for primary care, they involved different clinician groups and care settings across countries. AI-CDSSs evaluated in 1 primary care system may not perform similarly when implemented in health systems with different organizational structures or models of care, which may limit the transferability of these findings. Third, limitations related to the included studies constrain interpretation. The included studies were conducted exclusively in high-income countries, which limits the generalizability of these findings to low- and middle-income settings. The restriction to English and French publications may have limited the identification of relevant studies from other regions. This choice was made to ensure accurate data extraction and interpretation, as studies in other languages could not be reliably assessed by the review team. The classification of implementation contexts (simple, complicated, and complex) involved an element of interpretive judgment. To enhance transparency and reproducibility, we applied predefined criteria informed by the NASSS framework, including condition complexity, decision logic, system complexity, and preference sensitivity. Classification was performed using explicit decision rules and reviewer consensus. However, this approach remains dependent on the level of detail reported in primary studies, which was often limited, and may therefore not fully capture the underlying complexity of implementation contexts. Finally, evidence from the broader CDSS literature suggests that effectiveness may vary over time, with more recent systems showing modest improvements in clinician behavior [138]. However, we were unable to extract information on the development timeline, versioning, or updating procedures of AI models, as these data were not reported in the included studies. This limits the ability to determine whether observed effects reflect implementation context or differences related to the maturity of the systems evaluated.

### Conclusions

Available evidence on AI-CDSSs implemented in primary care demonstrates limited and inconsistent effectiveness, with safety poorly assessed. While positive effects were observed in simple clinical implementation contexts, these were primarily limited to clinician- and system-level outcomes. Improvements in outcomes were not consistent across more complex contexts and rarely translated into patient-level benefits. Safety conclusions are limited by inconsistent evaluation at the AI system level, patient level, and clinician level. The effectiveness conclusions are constrained by heterogeneity, short follow-up, and high or critical risk of bias. By synthesizing comparative evidence from clinically implemented AI-CDSSs across clinician-, patient-, and system-level outcomes and explicitly considering safety and implementation context, this review extends prior reviews that primarily mapped AI applications or implementation barriers in primary care. Clinical implementation research should evaluate safety as a multilevel construct and describe how implementation context shapes the use and impact of AI-CDSSs in clinical practice. Health care systems are adopting AI-CDSSs faster than they can ensure reliable and safe use, a gap demanding rigorous workflow integration, standardized evaluation frameworks, and systematic safety assessment to support trustworthy implementation in primary care.

## Supplementary material

10.2196/96882 Multimedia Appendix 1 Methodological specifications.

10.2196/96882 Multimedia Appendix 2 Databases search strategy.

10.2196/96882 Multimedia Appendix 3 Definitions and rules to classify the implementation contexts.

10.2196/96882 Multimedia Appendix 4 Mapping of included records to unique studies.

10.2196/96882 Multimedia Appendix 5 Detailed characteristics of the included studies.

10.2196/96882 Multimedia Appendix 6 Risk of bias assessment.

10.2196/96882 Multimedia Appendix 7 Characteristics of the participants.

10.2196/96882 Multimedia Appendix 8 Outcomes and effects.

10.2196/96882 Checklist 1 PRISMA 2020 checklist.

10.2196/96882 Checklist 2 PRISMA-S checklist

10.2196/96882 Checklist 3 SWiM checklist

## Abbreviations

AI-CDSS: AI-based clinical decision support system
CAT: Complexity Assessment Tool
CDSS: clinical decision support systems
CONSORT-AI: Consolidated Standards of Reporting Trials–AI
EHR: electronic health record
GRADE: Grading of Recommendations Assessment, Development, and Evaluation
NASSS: Non-Adoption, Abandonment, Scale-Up, Spread, and Sustainability
PICO: population, intervention, comparator, and outcomes
PRISMA: Preferred Reporting Items for Systematic Reviews and Meta-Analyses
PRISMA-S: Preferred Reporting Items for Systematic Reviews and Meta-Analyses literature search extension
RCT: randomized controlled trial
SWiM: Synthesis Without Meta-analysis
