# Supplementary material for: AI-Based Clinical Decision Support Systems in Primary Care: Systematic Review of Clinical Implementation
Source: J Med Internet Res. 2026 Oct 19;28:e96882. doi: 10.2196/96882 (PMC13679871; doi:10.2196/96882)
Supplement: Supplementary file 2 [file jmir-v28-e96882-s001.docx]

**Multimedia appendix 2: Post-registration methodological adaptations and rationale.**

*Operational specifications of prespecified methods.*

The following specifications operationalize methods prespecified in the registered protocol and do not represent departures from the planned review methods.

|  | Adaptation and rationale |
| --- | --- |
| RoB-2 | In the domain of risk of bias due to deviations from the intended interventions, we used the “effect of assignment to intervention” variant. This variant is recommended when the research question concerns the real-world effectiveness of the intervention (i.e., whether the AI-CDSS should be recommended for use in practice) which aligns with the review question. |
| ROBINS-I | Domain 1: We applied Variant A because all included studies enrolled clinicians and patients prospectively, and exposure status (AI-CDSS vs usual care) was defined at the start of follow-up.  Domain 4: We applied Variant A because deviations from intended use (e.g., non-use, partial use, overrides) occur during follow-up and can influence outcomes, which is the scenario Variant A is designed for. |

*Post-registration methodological deviations and adaptations.*

The following methodological adaptations occurred after protocol registration and are reported with their rationale in accordance with transparent systematic review reporting.

|  | Adaptation and rationale |
| --- | --- |
| RoB-ME | Although ROB-ME was prespecified in the protocol, it was not applied because the review did not produce a pooled meta-analytic estimate. ROB-ME is a synthesis-level tool designed to assess the risk of bias due to missing evidence in meta-analyses rather than individual study estimates. In the absence of sufficiently comparable outcome data to support meta-analysis, there was no synthesized estimate against which the tool could be meaningfully applied. |
| GRADE | Although GRADE assessments were prespecified for outcomes reported in at least two studies, they were not undertaken because the available evidence did not constitute sufficiently coherent bodies of evidence for meaningful certainty assessment. Most outcomes were reported in single studies, rendering the inconsistency domain (a core component of GRADE) unassessable. For the subset of outcomes reported in more than one study, substantial conceptual and methodological heterogeneity meant that studies could not be treated as addressing a sufficiently common question for indirectness and inconsistency judgments to be reliable or reproducible. Applying GRADE under these conditions would have resulted in certainty ratings driven primarily by structural features of the evidence rather than by a meaningful appraisal of a coherent body of evidence. |
| NASSS classification | Because substantial clinical and methodological heterogeneity limited interpretation of differences across studies, we introduced an exploratory classification of implementation contexts using an operationalized approach informed by selected items from the NASSS-CAT short version. This analysis was intended to provide a structured basis for interpreting differences in intervention effects across studies and did not alter the eligibility criteria, outcome definitions, or primary synthesis methods. |
