# Supplementary material for: AI-Based Clinical Decision Support Systems in Primary Care: Systematic Review of Clinical Implementation
Source: J Med Internet Res. 2026 Oct 19;28:e96882. doi: 10.2196/96882 (PMC13679871; doi:10.2196/96882)
Supplement: Supplementary file 3 [file jmir-v28-e96882-s002.docx]

**Multimedia appendix 4:** Databases search strategy

**Medline (OVID)**

**Date of the search:** 27-06-2025

**Database limit:** no database limit has been applied.

| **#** | **Search terms** | **Results** |
| --- | --- | --- |
| 1 | AI-CDSS.ti,ab,kf OR (AI adj2 ("Clinical Decision Support" OR CDS) adj3 system?).ti,ab,kf | 144 |
| 2 | Decision Support Systems, Clinical/ OR ("clinical decision support" adj3 (system? OR tool?)).ti,ab,kf | 13762 |
| 3 | exp Artificial Intelligence/ OR exp "Machine Learning"/ OR "Neural Networks, Computer"/ OR exp decision tree/ OR exp Classification Algorithms/ OR large language model?.ti,ab,kf OR natural language processing?.ti,ab,kf  OR (generative adj2 (AI OR model*)).ti,ab,kf OR ChatGPT?.ti,ab,kf OR "Chat GPT?".ti,ab,kf OR BERT.ti,ab,kf OR "support vector".ti,ab,kf OR "transformer model".ti,ab,kf OR "decision tree?".ti,ab,kf OR "random forest?".ti,ab,kf  OR (Bayes* adj2 network?).ti,ab,kf OR (predict* adj2 model*).ti,ab,kf OR ((artificial OR computational) adj2 intelligence).ti,ab,kf OR ((Machine? OR Supervised OR Unsupervised OR Reinforcement OR Deep OR predictive) adj2 Learning).ti,ab,kf OR "Neural Network?".ti,ab,kf OR "Classification Algorithm?".ti,ab,kf OR CNN.ti,ab,kf OR RNN.ti,ab,kf OR LLM.ti,ab,kf OR NLP.ti,ab,kf OR SVM.ti,ab,kf OR ((Ambient OR Clinical OR Medical OR automated OR virtual OR Digital OR AI OR "Artificial Intelligence" OR E) adj1 scrib*).ti,ab,kf | 641962 |
| 4 | Clinical Trial/ OR Controlled Clinical Trial/ OR Randomized Controlled Trial/ OR Evaluation Study/ OR Comparative Study/ OR Evaluation Studies as Topic/ OR Program Evaluation/ OR Random Allocation/ OR Non-Randomized Controlled Trials as Topic/ OR Controlled Before-After Studies/ OR Interrupted Time Series Analysis/ OR Multicenter Studies as Topic/ OR (multicenter OR multicentre OR "multi center" OR "multi centre").ti OR quasiexperiment*.ti,ab,kf OR "quasi experiment*".ti,ab,kf  OR random*.ti,ab,kf OR "double blind?".ti,ab,kf OR "controlled trial?".ti,ab,kf OR "control group".ti,ab,kf OR "clinical trial?".ti,ab,kf OR "interrupted time?".ti,ab,kf OR "time serie?".ti,ab,kf OR "time point*".ti,ab,kf OR pretest?.ti,ab,kf OR "pre test?".ti,ab,kf OR posttest?.ti,ab,kf OR "post test?".ti,ab,kf OR prepost?.ti,ab,kf OR "pre post?".ti,ab,kf OR "before and after".ti,ab,kf OR "repeated measur*".ti,ab,kf OR intervention?.ti,ab,kf OR Cohort Studies/ OR (Cohort adj2 (Stud* OR analys*)).ti,ab,kf | 6739182 |
| 5 | (1 OR (2 AND 3)) AND 4 | 1190 |

**Embase (Embase.com)**

**Date of the search:** 27-06-2025

**Database limit:** results have been limited to Embase database, prepublications & Clinical trials.

| **#** | **Search terms** | **Results** |
| --- | --- | --- |
| 1 | AI-CDSS:ti,ab,kw OR (AI NEAR/2 ("Clinical Decision Support" OR CDS) NEAR/3 system$):ti,ab,kw | 190 |
| 2 | 'decision support system'/de OR 'clinical decision support system'/de OR ("clinical decision support" NEAR/3 (system$ OR tool$)):ti,ab,kw | 41,762 |
| 3 | 'artificial intelligence'/exp OR 'generative artificial intelligence'/exp OR 'machine learning'/exp OR 'artificial neural network'/exp OR 'classification algorithm'/exp OR 'artificial intelligence chatbot'/exp OR 'support vector machine'/exp OR 'transformer model'/exp OR 'decision tree'/de OR 'Bayesian network'/de OR 'predictive model'/exp OR 'large language model'/exp OR "large language model$":ti,ab,kw OR "natural language processing$":ti,ab,kw OR (generative NEAR/2 (AI OR model*)):ti,ab,kw OR ChatGPT$:ti,ab,kw OR "Chat GPT$":ti,ab,kw OR BERT:ti,ab,kw OR "support vector":ti,ab,kw OR "transformer model":ti,ab,kw OR "decision tree$":ti,ab,kw  OR "random forest$":ti,ab,kw OR (Bayes* NEAR/2 network$):ti,ab,kw  OR (predict* NEAR/2 model*):ti,ab,kw OR ((artificial OR computational) NEAR/2 intelligence):ti,ab,kw OR ((Machine$ OR Supervised OR Unsupervised OR Reinforcement OR Deep OR predictive) NEAR/2 Learning):ti,ab,kw OR "Neural Network$":ti,ab,kw OR "Classification Algorithm$":ti,ab,kw OR CNN:ti,ab,kw OR RNN:ti,ab,kw OR LLM:ti,ab,kw OR NLP:ti,ab,kw OR SVM:ti,ab,kw OR ((Ambient OR Clinical OR Medical OR automated OR virtual OR Digital OR AI OR "Artificial Intelligence" OR E) NEAR/1 scrib*):ti,ab,kw | 979,151 |
| 4 | 'clinical trial'/de OR 'controlled clinical trial'/exp OR 'evaluation study'/exp OR 'comparative study'/de OR 'randomization'/de OR 'controlled clinical trial (topic)'/de OR 'intervention study'/de OR 'epidemiology'/de OR 'multicenter study (topic)'/de OR 'pretest posttest design'/de OR (multicenter OR multicentre OR "multi center" OR "multi centre"):ti OR quasiexperiment*:ti,ab,kw OR "quasi experiment*":ti,ab,kw OR random*:ti,ab,kw OR "double blind$":ti,ab,kw OR "controlled trial$":ti,ab,kw OR "control group":ti,ab,kw OR "clinical trial$":ti,ab,kw OR "interrupted time$":ti,ab,kw OR "time serie$":ti,ab,kw OR "time point$":ti,ab,kw OR pretest$:ti,ab,kw OR "pre test$":ti,ab,kw OR posttest$:ti,ab,kw OR "post test$":ti,ab,kw OR prepost$:ti,ab,kw OR "pre post$":ti,ab,kw OR "before and after":ti,ab,kw OR "repeated measur*":ti,ab,kw OR intervention$:ti,ab,kw  OR 'cohort analysis'/de OR (Cohort NEAR/2 (Stud* OR analys*)):ti,ab,kw | 9,298,144 |
| 5 | (#1 OR (#2 AND #3)) AND #4 | 4,035 |
| 6 | #5 AND [embase]/lim NOT ([embase]/lim AND [medline]/lim) | 1,414 |

**CINAHL**

**Date of the search:** 27-06-2025

**Database limit:** no database limit has been applied.

| **#** | **Search terms** | **Results** |
| --- | --- | --- |
| 1 | TI "AI-CDSS" OR AB "AI-CDSS" OR TI (AI N2 ("Clinical Decision Support" OR CDS) N3 system#) OR AB (AI N2 ("Clinical Decision Support" OR CDS) N3 system#) | 38 |
| 2 | MH "Decision Support Systems, Clinical" OR TI ("clinical decision support" N3 (system# OR tool#)) OR AB ("clinical decision support" N3 (system# OR tool#)) | 8,624 |
| 3 | MH "Artificial Intelligence, Generative+" OR MH "Artificial Intelligence+" OR MH "Machine Learning+" OR MH "Neural Networks (Computer)+" OR MH "Deep Learning+" OR MH "Prediction Algorithms" OR MH "Machine Learning Algorithms" OR MH "Classification Algorithms" OR MH "Prediction Models"  OR MH "Decision Trees+" OR TI "large language model#" OR TI "natural language processing#" OR TI (generative N2 (AI OR model*)) OR TI ChatGPT# OR TI "Chat GPT#" OR TI BERT OR TI "support vector" OR TI "transformer model" OR TI "decision tree#" OR TI "random forest#"  OR TI (Bayes* N2 network#) OR TI (predict* N2 model*) OR TI ((artificial OR computational) N2 intelligence) OR TI ((Machine# OR Supervised OR Unsupervised OR Reinforcement OR Deep OR predictive) N2 Learning) OR TI "Neural Network#" OR TI "Classification Algorithm#" OR TI CNN OR TI RNN  OR TI LLM OR TI NLP OR TI SVM OR TI ((Ambient OR Clinical OR Medical OR automated OR virtual OR Digital OR AI OR "Artificial Intelligence" OR E) N1 scrib*) OR AB "large language model#" OR AB "natural language processing#" OR AB (generative N2 (AI OR model*)) OR AB ChatGPT#  OR AB "Chat GPT#" OR AB BERT OR AB "support vector" OR AB "transformer model" OR AB "decision tree#" OR AB "random forest#"  OR AB (Bayes* N2 network#) OR AB (predict* N2 model*) OR AB ((artificial OR computational) N2 intelligence) OR AB ((Machine# OR Supervised OR Unsupervised OR Reinforcement OR Deep OR predictive) N2 Learning)  OR AB "Neural Network#" OR AB "Classification Algorithm#" OR AB CNN OR AB RNN OR AB LLM OR AB NLP OR AB SVM OR AB ((Ambient OR Clinical OR Medical OR automated OR virtual OR Digital OR AI OR "Artificial Intelligence" OR E) N1 scrib*) | 112,832 |
| 4 | MH "Clinical Trials" OR MH "Randomized Controlled Trials" OR MH "Pretest-Posttest Design+" OR MH "Nonrandomized Trials" OR MH "Interrupted Time Series Analysis" OR MH "Controlled Before-After Studies" OR MH "Evaluation Research" OR MH "Comparative Studies" OR MH "Program Evaluation"  OR MH "Random Assignment" OR MH "Multicenter Studies" OR TI (multicenter OR multicentre OR "multi center" OR "multi centre")  OR TI quasiexperiment* OR TI "quasi experiment*" OR TI random* OR TI "double blind#" OR TI "controlled trial#" OR TI "control group" OR TI "clinical trial#" OR TI "interrupted time#" OR TI "time serie#" OR TI "time point#" OR TI pretest# OR TI "pre test#" OR TI posttest# OR TI "post test#" OR TI prepost# OR TI "pre post#" OR TI "before and after" OR TI "repeated measur*" OR TI intervention# OR AB quasiexperiment* OR AB "quasi experiment*" OR AB random* OR AB "double blind#" OR AB "controlled trial#" OR AB "control group" OR AB "clinical trial#" OR AB "interrupted time#" OR AB "time serie#" OR AB "time point#" OR AB pretest# OR AB "pre test#" OR AB posttest# OR AB "post test#" OR AB prepost# OR AB "pre post#" OR AB "before and after" OR AB "repeated measur*" OR AB intervention# OR TI (Cohort N2 (Stud* OR analys*)) OR AB (Cohort N2 (Stud* OR analys*)) | 1,814,182 |
| 5 | (S1 OR (S2 AND S3)) AND S4 | 553 |

**Web of Science**

**Date of the search:** 27-06-2025

**Database limit:** no database limit has been applied.

| **#** | **Search terms** | **Results** |
| --- | --- | --- |
| 1 | TS="AI-CDSS" OR TS=(AI NEAR/2 ("Clinical Decision Support" OR CDS) NEAR/3 system$) | 193 |
| 2 | TS=("clinical decision support" NEAR/3 (system$ OR tool$)) | 7,900 |
| 3 | TS="large language model$" OR TS="natural language processing$" OR TS=(generative NEAR/2 (AI OR model*)) OR TS=ChatGPT$ OR TS="Chat GPT$" OR TS=BERT OR TS="support vector" OR TS="transformer model" OR TS="decision tree$" OR TS="random forest$" OR TS=(Bayes* NEAR/2 network$) OR TS=(predict* NEAR/2 model*) OR TS=((artificial OR computational) NEAR/2 intelligence) OR TS=((Machine$ OR Supervised OR Unsupervised OR Reinforcement OR Deep OR predictive) NEAR/2 Learning)  OR TS="Neural Network$" OR TS="Classification Algorithm$" OR TS=CNN  OR TS=RNN OR TS=LLM OR TS=NLP OR TS=SVM OR TS=((Ambient OR Clinical OR Medical OR automated OR virtual OR Digital OR AI OR "Artificial Intelligence" OR E) NEAR/1 scrib*) | 2,476,093 |
| 4 | TI=(multicenter OR multicentre OR "multi center" OR "multi centre") OR TS=quasiexperiment* OR TS="quasi experiment*" OR TS=random* OR TS="double blind$" OR TS="controlled trial$" OR TS="control group" OR TS="clinical trial$" OR TS="interrupted time$" OR TS="time serie$" OR TS="time point$" OR TS=pretest$ OR TS="pre test$" OR TS=posttest$ OR TS="post test$" OR TS=prepost$ OR TS="pre post$" OR TS="before and after" OR TS="repeated measur*" OR TS=intervention$  OR TS=(Cohort NEAR/2 (Stud* OR analys*)) | 6,657,880 |
| 5 | (#1 OR (#2 AND #3)) AND #4 | 750 |

**CENTRAL**

**Date of the search:** 27-06-2025

**Database limit:** no database limit has been applied.

| **#** | **Search terms** | **Results** |
| --- | --- | --- |
| 1 | AI-CDSS:ti,ab,kw OR (AI adj2 ("Clinical Decision Support" OR CDS) NEAR/3 system?):ti,ab,kw | 10 |
| 2 | [mh "Decision Support Systems, Clinical"] OR ("clinical decision support" NEAR/3 (system? OR tool?)):ti,ab,kw | 1313 |
| 3 | [mh "Artificial Intelligence"] OR [mh "Machine Learning"] OR [mh ^"Neural Networks, Computer"] OR [mh "decision tree"] OR [mh "Classification Algorithms"] OR "natural language processing?":ti,ab,kw OR (generative NEAR/2 (AI OR model OR models OR modelling)):ti,ab,kw OR ChatGPT?:ti,ab,kw OR "Chat GPT?":ti,ab,kw OR BERT:ti,ab,kw OR "support vector":ti,ab,kw OR "transformer model":ti,ab,kw OR "decision tree?":ti,ab,kw  OR "random forest?":ti,ab,kw OR (Bayes* NEAR/2 network?):ti,ab,kw  OR (predict* NEAR/2 (model OR models OR modelling)):ti,ab,kw OR ((artificial OR computational) NEAR/2 intelligence):ti,ab,kw OR ((Machine? OR Supervised OR Unsupervised OR Reinforcement OR Deep OR predictive) NEAR/2 Learning):ti,ab,kw OR "Neural Network?":ti,ab,kw OR "Classification Algorithm?":ti,ab,kw OR CNN:ti,ab,kw OR RNN:ti,ab,kw OR LLM:ti,ab,kw  OR NLP:ti,ab,kw OR SVM:ti,ab,kw OR ((Ambient OR Clinical OR Medical OR automated OR virtual OR Digital OR AI OR "Artificial Intelligence" OR E) NEAR/1 scribe):ti,ab,kw | 17312 |
| 4 | (#1 OR (#2 AND #3)) | 168 |
| 5 | *Trials database limit* | 167 |
