# Supplementary material for: AI-Based Clinical Decision Support Systems in Primary Care: Systematic Review of Clinical Implementation
Source: J Med Internet Res. 2026 Oct 19;28:e96882. doi: 10.2196/96882 (PMC13679871; doi:10.2196/96882)
Supplement: Supplementary file 4 [file jmir-v28-e96882-s003.docx]

**Multimedia appendix 6:** Definitions and rules to classify the implementation contexts.

*Condition complexity* captured whether the clinical problem was narrow and homogeneous or heterogeneous with variable trajectories. *Clinical decision logic* was classified as protocolized when decisions could be guided by explicit rules, thresholds, or guideline-based triggers; structured when decisions relied on probabilistic estimates or algorithmic guidance within a constrained clinical pathway; and individualized when decisions required integration of heterogeneous clinical information that could not be readily reduced to standardized decision rules. *System complexity* was defined as operationally simple when AI-CDSS outputs were generated from routinely available clinical data and integrated into care with minimal additional workflow steps; structured when clinicians were required to review additional interface components such as dashboards, predicted outcomes, or comparative visualizations; and multi-component when the system required additional data collection, input, or iterative processes involving clinicians and/or patients before use in care. Preference sensitivity was added because AI-CDSS directly support clinical decisions, and the expected impact of such systems may differ depending on whether the decision has one dominant guideline-concordant option or requires trade-offs based on patient values, goals, tolerance, and context [1]. *Preference sensitivity* was operationalized as low when one clinically dominant option existed or when multiple options were strongly guided by established recommendations; moderate when decisions involved trade-offs within a constrained set of clinically acceptable options; and high when decisions were primarily shaped by patient values, goals, tolerance, or contextual considerations.

**Rating:**

| Authors | Condition complexity | Clinical decision logic | System complexity | Preference sensitivity | **Overall** |
| --- | --- | --- | --- | --- | --- |
| Alcoceba-Herrero et al. |  |  |  |  | **SIMPLE** |
| Areias et al. |  |  |  |  | **COMPLEX** |
| Chen et al. |  |  |  |  | **SIMPLE** |
| Cruz et al. |  |  |  |  | **COMPLICATED** |
| Granviken et al. |  |  |  |  | **COMPLEX** |
| Herter et al. |  |  |  |  | **COMPLICATED** |
| Liu et al. |  |  |  |  | **SIMPLE** |
| Popescu et al.  Qassim et al. |  |  |  |  | **COMPLEX** |
| Romero-Brufau et al. |  |  |  |  | **COMPLICATED** |
| Seol et al. |  |  |  |  | **COMPLICATED** |
| Yao et al.  Rushlow et al.  Thao et al. |  |  |  |  | **SIMPLE** |

REFERENCE:

1.	Keirns CC, Goold SD. Patient-Centered Care and Preference-Sensitive Decision Making. JAMA. 2009;302(16):1805-6. doi: 10.1001/jama.2009.1550.
