# Supplementary material for: AI-Based Clinical Decision Support Systems in Primary Care: Systematic Review of Clinical Implementation
Source: J Med Internet Res. 2026 Oct 19;28:e96882. doi: 10.2196/96882 (PMC13679871; doi:10.2196/96882)
Supplement: Supplementary file 5 [file jmir-v28-e96882-s004.docx]

**Multimedia appendix 7**: Mapping of included records to unique studies.

| Study label | All associated records | n | Additional information |
| --- | --- | --- | --- |
| Alcoceba-Herrero et al. | Alcoceba-Herrero et al | 1 | Primary report |
| Areias et al. | Areias et al. | 1 | Primary report |
| Chen et al. | Chen et al. | 1 | Primary report |
| Cruz et al. | Cruz et al. | 1 | Primary report |
| Granviken et al. | Granviken et al. | 1 | Primary report |
| Herter et al. | Herter et al. | 1 | Primary report |
| Liu et al. | Liu et al. | 1 | Primary report |
| Popescu et al. | Popescu et al.  Qassim et al. | 2 | Primary report (quantitative evaluation)  Secondary report (qualitative evaluation) |
| Romero-Brufau et al. | Romero-Brufau et al. | 1 | Primary report |
| Seol et al. | Seol et al. | 1 | Primary report |
| Yao et al. | Yao et al.  Rushlow et al.  Thao et al. | 3 | Primary report (quantitative evaluation)  Secondary report (adoption evaluation)  Secondary report (economic evaluation) |
