# Supplementary material for: AI-Based Clinical Decision Support Systems in Primary Care: Systematic Review of Clinical Implementation
Source: J Med Internet Res. 2026 Oct 19;28:e96882. doi: 10.2196/96882 (PMC13679871; doi:10.2196/96882)
Supplement: Supplementary file 6 [file jmir-v28-e96882-s005.docx]

**Multimedia appendix 8:** Detailed characteristics of the included studies.

| Author  *Year of publication*  Country of recruitment | Study design | Study period | Changes to methods after trial commencement | Presence of conflicts of interests | Sources of funding |
| --- | --- | --- | --- | --- | --- |
| Alcoceba-Herrero et al.  *2024*  Spain | Prospective, multicenter, open-label RCT | November 2021 to January 2022 | Not reported | No | ·Academic |
| Areias et al.  *2024*  *USA* | Post hoc analysis of a prospective, pre-post cohort study | Control period: 18 June 2020 to 13 June 2022  Intervention period: 21 November 2022 to 8 July 2023 | Not reported | Yes | ·Industrial  ·Governmental |
| Chen et al.  *2020*  *USA* | Pilot study (biweekly on/off cycles) | January 19 to March 11, 2019 | Not reported | No | ·Not reported |
| Cruz et al.  2019  *Spain* | Not reported (assumed as quasi-experimental before-after study) | ·Initial evaluation:  01/01/2016 to  31/05/2017  ·Postimplementation assessment:  27/11/2017 to  31/12/2017 | Not reported | Not reported | ·Not reported |
| Granviken et al.  2025  *Norway* | A cluster RCT | February 2021 to November 2021 | Not reported | No | ·Governmental |
| Herter et al.  2022  *Netherlands* | Prospective observational study | 4 months, starting in November 2017 | Not reported | Yes | ·Industrial |
| Liu et al.  2021  *USA* | Prospective cohort study | January 1, 2018, to August 31,  2018 | Not reported | Yes | ·Industrial |
| Popescu et al.  2021  Qassim et al.  2023  *Canada* | Single-arm, naturalistic, longitudinal feasibility study; follow-up study | January to November 2020 | Adaptation to be completed entirely via telemedicine due to COVID-19 | Yes | ·Industrial  ·Governmental |
| Romero-Brufau et al.  2020  *USA* | Pre-post survey-based quality improvement study | ·Pre-intervention: September 2018  ·Post-intervention: February 2019. The go-live date for the implementation was in November 2018. | Not reported | No | ·Supported in part by internal institutional research funds from Mayo Clinic, a nonprofit academic medical center. |
| Seol et al.  2021  *USA* | A single-center pragmatic RCT with a stratified randomization design | December 13, 2016, to December 12, 2017 | Not reported | No | ·Industrial  ·Governmental |
| Yao et al.  2021  Rushlow et al.  2022  Thao et al.  2024  *USA* | 1) Cluster RCT; 2) secondary analysis; and 3) model-based cost-effectiveness analysis (decision tree + lifetime Markov model) using secondary data | August 5, 2019,  and March 31,  2020 | Not reported | Yes | ·Academic/institutional, internal to Mayo Clinic. |

***RCT***: Randomized Controlled Trial
