# Supplementary material for: AI-Based Clinical Decision Support Systems in Primary Care: Systematic Review of Clinical Implementation
Source: J Med Internet Res. 2026 Oct 19;28:e96882. doi: 10.2196/96882 (PMC13679871; doi:10.2196/96882)
Supplement: Supplementary file 8 [file jmir-v28-e96882-s007.docx]

**Multimedia appendix 10**: Characteristics of the participants

| Authors | Clinicians: eligibility criteria | Clinicians: sample size and baseline characteristics | Participants: eligibility criteria | Participants: sample size and baseline characteristics |
| --- | --- | --- | --- | --- |
| Alcoceba-Herrero et al.  *2024*  Spain | Not reported | Not reported | Inclusion: a confirmed COVID-19 diagnosis by PCR or antigen test within ≤6 days of study inclusion, home internet access, possession of an Android smartphone, ability to electronically sign informed consent, and willingness to wear monitoring devices if assigned to the case group.  Exclusion: age under 16 years, insufficient digital skills to use the home app, hospitalization or clinical progression likely to require admission between diagnosis and inclusion, and disabling upper limb or cognitive impairments preventing participation. | Experimental:  ·n=170  ·Age:  <30: 12.6%  30-45: 37.9%  46-65: 44.7%  >65: 4.9%  ·Gender: 49.5% woman  ·COVID-19 PCR test positive: 67.0%  ·COVID-19 antigen test positive: 33.0%  ·COVID-19 vaccinated: 67.0%  ·High blood pressure: 9.7%  ·Diabetes mellitus: 2.9%  ·Obesity: 9.7%  ·Dyslipidemia: 16.5%  ·Atrial fibrillation: 1.0%  ·Myocardial infarction: 0.0%  ·Asthma: 7.8%  ·Bronchitis: 0.0%  ·COPD: 1.9%  ·Stroke: 0.0%  ·Liver disease: 1.0%  ·Neoplasia: 0.0%  ·Chronic renal failure: 0.0%  ·Corticosteroids: 2.9%  ·Immunosuppressive biological therapy: 0.0%    Control:  ·n=172  ·Age:  <30: 19.4%  30-45: 38.2%  46-65: 39.6%  >65: 2.8%  ·Gender: 62.5% woman  ·COVID-19 PCR test positive: 66.7%  ·COVID-19 antigen test positive: 33.3%  ·COVID-19 vaccinated: 70.8%  ·High blood pressure: 7.6%  ·Diabetes mellitus: 2.1%  ·Obesity: 9.0%  ·Dyslipidemia: 10.4%  ·Atrial fibrillation: 2.8%  ·Myocardial infarction: 0.7%  ·Asthma: 6.9%  ·Bronchitis: 0.7%  ·COPD: 0.0%  ·Stroke: 2.1%  ·Liver disease: 0.0%  ·Neoplasia: 2.8%  ·Chronic renal failure: 0.7%  ·Corticosteroids: 3.5%  ·Immunosuppressive biological therapy: 3.5% |
| Areias et al.  *2024*  *USA* | Not reported | Not reported | Inclusion: the beneficiaries of employer health plans who reported chronic musculoskeletal pain (defined as persistent or recurrent pain lasting ≥ 12 weeks) in the ankle, elbow, hip, knee, low back, neck, shoulder, wrist, or hand were invited to participate in the study through a dedicated enrollment website.  Exclusion: (1) health conditions that were incompatible with at least 20 min of light to moderate exercise; (2) ongoing cancer treatment; and (3) the presence of signs or symptoms indicative of serious pathology (e.g., rapid progressive motor weakness, sensory alterations, or bowel or bladder dysfunction). | Post-implementation (experimental):  ·n=24083  ·Mean age= 48.5 (SD=11.6)  ·Sex: 58.7% woman  ·Ethnicity:  Asian: 9.8%  Black: 9.8%  Hispanic: 9.6%  Non-Hispanic White: 67.9%  Other: 2.9%  ·Mean pain intensity: 4.73 (SD=1.9)  Pre-implementation (control):  ·n=12103  ·Mean age= 50.0 (SD=11.7)  ·Gender: 57.9% woman  ·Ethnicity:  Asian: 10.2%  Black: 10.8%  Hispanic: 9.8%  Non-Hispanic White: 66.3%  Other: 2.8%  ·Mean pain intensity: 4.84 (SD=1.9) |
| Chen et al.  *2020*  *USA* | Not reported | Not reported | Not reported | Not reported |
| Cruz et al.  2019  *Spain* | Not reported | ·n=86  ·Profession:  Physicians  Pediatricians | Not reported | Not reported |
| Granviken et al.  2025  *Norway* | Not reported | Experimental:  ·n=22 physiotherapists  ·Mean age= 39.7 (SD=7.9)  ·Sex: 31.8% female  Experience as a PT: mean=15.3 years (SD=7.6)  Specialty as a manual therapist: 27.3%  Control:  ·n=22 physiotherapists  ·Mean age= 40.8 (SD=8.8)  ·Sex: 27.3% female  Experience as a PT: mean=15.4 years (SD=8.6)  Specialty as a manual therapist: 27.3% | Inclusion criteria : Patients 18 years and older presenting to the physiotherapist with clinician determined MSK pain in the shoulder, neck, upper/low back, hip, knee, or with multisite/ complex pain of any duration as the main reason for seeking physiotherapy were included. Aligned with The International Association of Pain classification of chronic pain for ICD-11, we included patients with chronic primary pain as the index diagnosis. Chronic secondary MSK pain could be a comorbidity, but not an index diagnosis.  Exclusion criteria: cognitive function or insufficient skills in the Norwegian language, ongoing cancer, pregnancy or pregnancy-related conditions, surgery or fracture within the last 6 months, patients scheduled for surgery, or neurological diagnosis. | Experimental:  ·n=358  ·Mean age= 47.0 (SD=14.9)  ·Sex: 66.5% female  ·Pain duration:  <3 months: 24.0%  3-12 months: 24.0%  >12 months: 52.0%  ·BMI: 26.4 (SD=5.2)  ·Married or living with partner: 67.8%  ·Education:  Primary/high school 38.7%  Up to 4y of higher education 29.1%  4y or more of higher education: 27.5%  Other: 4.8%  ·Employment status:  Sick leave 12.6%  Disability pension or work assessment 13.1%  Paid employment 51.1%  Retired 12.3%  Student 9.2%  Other 1.7%  ·Comorbidity  No comorbidity 36.6%  1 comorbidity 30.7%  2-3 comorbidities 26.8%  4 or more comorbidities 5.9%  ·Primary pain location:  Neck 13.6%  Shoulder 16.7%  Back 22.3%  Hip 12.1%  Knee 17.8%  Complex 17.5%  ·STart MSK Tool:  Low 36.7%  Medium 50.7%  High 12.6%  ·OMSPQ (0-100) mean= 43.8 (SD=15.7)  ·Anxiety for pain in physical activity (0-10): mean=3.7 (SD=3.1)  ·Physical inactivity: 27.4%  ·Impaired health literacy: 17.6%  ·Baseline measures of outcomes:  PSFS (0-10), mean = 3.8 (SD=2.3)  Pain last week (0-10), mean=4.7 (SD=2.2)  Work ability (0-10), mean =6.0 (SD=3.0)  PSEQ (0-12), mean=9.1 (SD=2.9)  HSCL-10 (1.0-4.0), mean=1.69 (SD=0.55)  MSK-HQ (0-56), mean= 35.9 (SD=9.0)  Sleep problems (%), 39.4%  Vitality problems 37.2%  Number of pain sites, mean = 3.5 (SD=2.2)  Use of pain medication last week, 58.3%  EQ5D index, mean = 0.75 (SD=0.18)  Control:  ·n=366  ·Mean age= 49.8 (SD=15.1)  ·Sex: 61.7% female  ·Pain duration:  <3 months: 23.5%  3-12 months: 27.3%  >12 months: 49.2%  ·BMI: 26.7 (SD=4.9)  ·Married or living with partner: 71.5%  ·Education:  Primary/high school 38.7%  Up to 4y of higher education 32.4%  4y or more of higher education: 23.6%  Other: 5.2%  ·Employment status:  Sick leave 10.9%  Disability pension or work assessment 14.2%  Paid employment 51.9%  Retired 14.5%  Student 6.8%  Other 1.6%  ·Comorbidity  No comorbidity 33.6%  1 comorbidity 34.4%  2-3 comorbidities 27.3%  4 or more comorbidities 4.6%  ·Primary pain location:  Neck 15.7%  Shoulder 18.7%  Back 18.2%  Hip 11.0%  Knee 18.5%  Complex 17.9%  ·STart MSK Tool:  Low 35.9%  Medium 50.3%  High 13.8%  ·OMSPQ (0-100) mean= 43.8 (SD=15.0)  ·Anxiety for pain in physical activity (0-10): mean=3.5 (SD=3.1)  ·Physical inactivity: 29.2%  ·Impaired health literacy: 13.1%  ·Baseline measures of outcomes:  PSFS (0-10), mean = 4.2 (SD=2.3)  Pain last week (0-10), mean=4.8 (SD=2.2)  Work ability (0-10), mean =6.1 (SD=2.8)  PSEQ (0-12), mean=9.2 (SD=3.0)  HSCL-10 (1.0-4.0), mean=1.63 (SD=0.48)  MSK-HQ (0-56), mean= 36.2 (SD=8.7)  Sleep problems (%), 35.7%  Vitality problems 37.0%  Number of pain sites, mean = 3.4 (2.1)  Use of pain medication last week, 54.8%  EQ5D index, mean = 0.77 (SD=0.17) |
| Herter et al.  2022  *Netherlands* | Not reported | Not reported | Inclusion: ≥1 indication of UTI symptoms (specific ICPC codes) + prescribed antibiotic for UTI, age >12 years. | During implementation  Experimental group:  ·n=3422 with uncertainty of AI-CDSS use  ·88% female  ·Age:  <30: 17%  30-50: 21%  50-70: 36%  >70: 26%  ·Diabetes: 8%  ·UTI with tissue invasion: 11%  ·Complicated UTI: 12%  ·n = 1121 with certainty of AI-CDSS use  ·92% female  ·Age:  <30: 15%  30-50: 18%  50-70: 39%  >70: 28%  ·Diabetes: 9%  ·UTI with tissue invasion: 13%  ·Complicated UTI: 13%  Control group:  ·n=3360  ·86% female  ·Age:  <30: 17%  30-50: 20%  50-70: 29%  >70: 33%  ·Diabetes: 13%  ·UTI with tissue invasion: 12%  ·Complicated UTI: 13% |
| Liu et al.  2021  *USA* | Not reported | Not reported | Inclusion criteria: Patients with type 1 or type 2 diabetes mellitus older than 18 years who had not attended an ophthalmology appointment within 1 year of the screening visit. Exclusion criteria: not able to speak English or had a history of seizure disorders. | Prospective cohort (experimental):  ·n=180  ·Age: mean=57.4 (SD=11.4)  ·Gender: 53.9% female  ·Race:  White: 15.0%  Black: 78.3%  Other: 5.6%  Unknown: 1.1%  ·Glycated hemoglobin index (n=140): 8.4 (2.1)  Historical cohort (control):  ·n=974  ·Age: mean=56.0 (SD=13.8)  ·Gender: 58.0% female  ·Race:  White: 16.5%  Black: 76.6%  Other: 6.7%  Unknown: 0.2%  ·Glycated hemoglobin index (n=763): 8.0 (2.1) |
| Popescu et al.  2021  Qassim et al.  2023  *Canada* | Inclusion criterion: family physicians or psychiatrists treating patients with depression on at least a monthly basis. | Participating clinicians were family physicians and psychiatrists practicing in Québec hospitals or clinics, all experienced in treating major depressive disorder on at least a monthly basis.  The clinician cohort included seven participating physicians out of ten initially recruited, with three unable to enroll patients due to COVID-19–related disruptions. No missing follow-up data for participating clinicians were reported, and their involvement spanned the full study period from January to November 2020.  ·Profession:  n=4 general practitioners  n=3 psychiatrists | Inclusion criteria: patients of enrolled physicians who were aged at least 18 years and diagnosed with an active major depression by  the physician as per Diagnostic and Statistical Manual of Mental  Disorders, Fifth Edition (DSM-5) criteria, able and willing to provide informed consent, and not diagnosed (or suspected) with bipolar affective disorder, as per DSM-5 criteria. Patients were required to be physically and mentally able to use a  computer or smartphone (ie, to not be delirious or have sufficient  cognitive function) but not necessarily to already be adept at it. | ·n=14 patients  ·Mean age: 36.43 (SD=14.84)  ·Sex: 64.29% female  ·Gender: 64.29% woman  ·Ethnicity:  Caucasian: 76.92%  Caribbean: 7.69%  African or African American: 7.69%  ·Residency status:  Canadian citizen 84.62%  Immigrant status (>5 years ago) 7.69%  Immigrant status (<5 years ago) 7.69%  ·Adoption status:  Adopted (7.69%, n=1)  ·Employment status:  Full-time 53.85 % (n=7)  Part-time 7.69% (n=1)  Disability (not working) 15.38% (n=2)  Unemployed + volunteer work 15.38% (n=2)  Unemployed 7.69% (n=1)  ·Relationship status  Married 30.77% (n=4)  Divorced 7.69% (n=1)  Dating a single partner 15.38% (n=2)  Not in a relationship 46.15% n=6)  ·Years of education: mean=15 (SD=6.42)  ·Income (CAD): mean 82,333.33 (SD=70,099.93) |
| Romero-Brufau et al.  2020  *USA* | Not reported | n=81  Pre-implementation:  ·n=45  ·Profession:  n=14 physicians  n=1 advanced practice provider  n=12 nurses  n=12 clinical assistants  n=6 other  Post-implementation:  ·n=38  ·Profession:  n=7 physicians  n=0 advanced practice provider  n=21 nurses  n=4 clinical assistants  n=3 other  n=3 missing data | Inclusion criterion: Patients with diabetes | Not reported |
| Seol et al.  2021  *USA* | Not reported | ·n=42  ·Profession:  Residents  Primary care providers | Inclusion criteria: children 1) under age 18 living in Olmsted County, 2) who had received medical care from pediatric primary care practice, Mayo Clinic, Rochester, between 2013 and 2016 and 3) research authorization using medical record for research. | Experimental:  ·n=90  ·Median age: 9.3 (IQR= 4.8 ; 13.3)  ·Sex: 41% female  ·Race:  White: 76%  ·Age group:  0-4 years: 25 (28%)  5-11: 32 (36%)  12-17: 33 (37%)  ·Stratum:  Stratum 1: 38 (42%)  Stratum 2: 23 (26%)  Stratum 3: 29 (32%)  ·HOUSES (An individual-level socioeconomic status measures based on real property data)  Q1: 7 (8%)  Q2: 21 (25%)  Q3: 33 (39%)  Q4: 23 (27%)  ·Living in high traffic volume: 15 (17%)  ·Availability of patient online portal: 77 (86%)  ·Family history of asthma: 50 (56%)  ·History of eczema: 41 (46%)  ·History of allergic rhinitis: 37 (41%)  ·Seasonal influenza vaccine: 68 (76%)  ·Discussion asthma at the general medical exam: 26/47 (55%)  ·Health care cost: median=968, IQR=538-1958  ·History of asthma exacerbation: 15 (17%)  ·High-risk by prediction score 36 (40%)  ·Primary Care Providers:  Residents: 26 (29%)  Other: 64 (71%)  Control:  ·n=94  ·Median age: 8.3 (IQR= 5.4 ; 12.7)  ·Sex: 46% female  ·Race:  White: 68%  ·Age group:  0-4 years: 19 (20%)  5-11: 49 (52%)  12-17: 26 (28%)  ·Stratum:  Stratum 1: 38 (40%)  Stratum 2: 30 (32%)  Stratum 3: 26 (28%)  ·HOUSES (An individual-level socioeconomic status measures based on real property data)  Q1: 15 (17%)  Q2: 26 (30%)  Q3: 19 (22%)  Q4: 27 (31%)  ·Living in high traffic volume: 14 (15%)  ·Availability of patient online portal: 75 (80%)  ·Family history of asthma: 40 (43%)  ·History of eczema: 47 (50%)  ·History of allergic rhinitis: 43 (46%)  ·Seasonal influenza vaccine: 81 (86%)  ·Discussion asthma at the general medical exam: 24/44 (55%)  ·Health care cost: median=932, IQR=460-1918  ·History of asthma exacerbation: 23 (25%)  ·High-risk by prediction score 41 (44%)  ·Primary Care Providers:  Residents: 29 (31%)  Other: 65 (69%) |
| Yao et al.  2021  Rushlow et al.  2022  Thao et al.  2024  *USA* | Inclusion criteria: primary care clinicians, including physicians, nurse practitioners and physician assistants, organized as teams that care for adult patients (≥18 years).  Exclusion criteria: Clinicians in the pediatric care teams, resident care teams, acute and urgent care teams, and nursing home care teams were excluded. | Experimental:  ·n=181  ·Specialty:  n=131 family medicine,  n=49 internal medicine,  n=1 other  ·Profession:  n=117 physicians,  n=22 physician assistants,  n=42 nurse practitioners  ·Mean age: 44.8 (SD=10.5)  ·Sex: 57.6% female  ·Race:  White: 93.2%  Black of African American: 1.2%  Asian: 3.7%  Other: 1.9%  ·Ethnicity:  Hispanic or Latino: 1.8%  Years in practice: mean=13.0 (10.25) [1.0; 43.0]  Years in current care team: Mean=8.6 (8.68) [0.0, 43.0]  Over the past 12 months, approximately what percent of your time do you work in direct patient care (include inpatient, outpatient and supervisory roles)?, n (%):  less than 25%: 3 (1.7%)  25-50%: 8 (4.7%)  51-75%: 23 (13.4%)  76-100%: 138 (80.2%)  Missing data: 9  How comfortable are you managing patients with LV dysfunction?, n (%) :  Not comfortable at all (1): 8 (4.7%)  (2) 15 (8.8%)  Somewhat comfortable (3) 51 (30.0%)  (4) 67 (39.4%)  Completely comfortable (5): 29 (17.1%)  Missing 11  How often do you consult cardiology for the management of LV dysfunction?, n (%) :  Never (1): 3 (1.7%)  (2): 51 (29.7%)  (3) Somewhat often: 74 (43.0%)  (4) 28 (16.3%)  (5) Always: 16 (9.3%)  Missing 9  Control:  ·n=177  ·Specialty:  n=127 family medicine,  n=49 internal medicine,  n=1 other  ·Profession:  n=118 physicians,  n=21 physician assistants,  n=38 nurse practitioners  ·Mean age: 46.1 (SD=11.01)  ·Sex: 59.7% female  ·Race:  White: 90.3%  Black of African American: 1.8%  Asian: 6.7%  Other: 1.2%  ·Ethnicity:  Hispanic or Latino: 1.7%  Years in practice: mean=14.2 (10.65) [0.5; 40.0]  Years in current care team: Mean=8.8 (8.60) [0.0, 40.0]  Over the past 12 months, approximately what percent of your time do you work in direct patient care (include inpatient, outpatient and supervisory roles)?, n (%):  less than 25%: 4 (2.3%)  25-50%: 8 (4.5%)  51-75%: 36 (20.5%)  76-100%: 128 (72.7%)  Missing data: 1  How comfortable are you managing patients with LV dysfunction?, n (%) :  Not comfortable at all (1): 7 (4.0%)  (2) 16 (9.1%)  Somewhat comfortable (3) 52 (29.5%)  (4) 72 (40.9%)  Completely comfortable (5): 29 (16.5%)  Missing 1  How often do you consult cardiology for the management of LV dysfunction?, n (%) :  Never (1): 0 (0.0%)  (2): 44 (25.0%)  (3) Somewhat often: 77 (43.8%)  (4) 36 (20.5%)  (5) Always: 19 (10.8%)  Missing 1 | Exclusion criteria: Patients were excluded if they were under 18 years old, had known EF ≤ 50% or a history of documented HF identified by clinical notes abstracted by natural language processing (NLP) or they did not provide authorization to use their data for research. | Experimental:  ·n=11573  ·Mean age: 60.5 (SD=17.5)  ·Sex: 52.5% female  ·Race:  White: 94.4%  Black of African American: 1.7%  Asian: 1.3%  Other: 2.2%  ·Age categories:  18-64: 54.1%  65-74: 23.9%  >=75: 22.1%  ·Rural: 54.6%  ·Medical history:  Hypertension: 56.1%  Diabetes: 20.3%  Myocardial infarction: 6.7%  Peripheral artery disease: 3.6%  Stroke or TIA: 3.5%  Prior AF: 8.6%  New AF on index ECG: 2.1%  Valvular heart disease: 1.1%  Chronic kidney disease: 11.9%  Prior echocardiogram: 16.4%  ·Location of ECG ordered:  Outpatient clinic: 52.2%  Emergency room: 38.1%  Hospital: 9.7%  Control:  ·n=11068  ·Mean age: 60.5 (SD=17.6)  ·Sex: 55.3% female  ·Race:  White: 94.2%  Black of African American: 1.9%  Asian: 1.2%  Other: 2.1%  ·Age categories:  18-64: 53.6%  65-74: 23.8%  >=75: 22.6%  ·Rural: 45.4%  ·Medical history:  Hypertension: 55.8%  Diabetes: 20.1%  Myocardial infarction: 6.5%  Peripheral artery disease: 4.0%  Stroke or TIA: 3.4%  Prior AF: 8.3%  New AF on index ECG: 2.2%  Valvular heart disease: 1.4%  Chronic kidney disease: 10.9%  Prior echocardiogram: 17.1%  ·Location of ECG ordered:  Outpatient clinic: 53.9%  Emergency room: 36.6%  Hospital: 9.4% |
