# Supplementary material for: AI-Based Clinical Decision Support Systems in Primary Care: Systematic Review of Clinical Implementation
Source: J Med Internet Res. 2026 Oct 19;28:e96882. doi: 10.2196/96882 (PMC13679871; doi:10.2196/96882)
Supplement: Supplementary file 9 [file jmir-v28-e96882-s008.docx]

**Multimedia appendix 11:** Outcomes and effects

| Authors | Outcomes  (measurement instruments if applicable) | Results |
| --- | --- | --- |
| Alcoceba-Herrero et al. | ·Number of alerts received and the ability of the system to resolve them autonomously | **Number of symptom alerts:**  ·Control: 1825 ; Experimental: 1209 (p < 0.05 in favor of experimental)  **Number of monitoring alerts (only experimental group):**  ·60618 alerts:  -57657 (95%) were technological alerts resolved autonomously by the AI-CDSS  -2961 (5%) were medical alerts managed by physicians.  ·Of the 2,961 medical alerts:  -2556 were mild or moderate and were managed by healthcare staff.  -405 were severe and were automatically escalated to EHS once they were confirmed by the patient. This happened in five cases. The remaining 400 severe alerts (not confirmed by the patient) were managed by clinicians by checking on the system’s dashboard that they were transitory alterations that did not require additional support. In this way, there were no false positives in terms of serious alerts resulting in unnecessary escalation to the EHS. |
| Areias et al. | ·Pain intensity  (NPRS)  ·Pain response rate  (≥30% change in pain intensity)  ·Mental health  (GAD-7 for anxiety, PHQ-9 for depression)  ·Engagement  (completion rate, session frequency, communication metrics)  ·PT-to-patient ratios  (Calculated monthly as the average number of patients per PT, regardless of treatment stage)  ·Satisfaction with the program  (0–10 likelihood to recommend)  ·Safety  (adverse event rate) | **Pain intensity:**  **·** Control: -1.9 [-2.0 ; -1.8] ; Experimental: -1.9 [-1.9 ; -1.8] (p=0.012 in favor of control but not meaningful)  **Pain response rate:**  ·Control: 63% [61 ; 64] ; Experimental: 64% [62 ; 65] (p=0.399. effect size=0.01)  **Mental health:**  ·Anxiety mean change:  Control: -4.1 [-4.4 ; -3.9] ; Experimental: -4.0 [-4.2 ; -3.7] (p=0.928, effect size=-0.04)  ·Depression mean change:  Control: -4.7 [-5.1 ; -4.3] ; Experimental: -4.0 [-4.3 ; -3.7] (p=0.187, effect size=-0.30)  **Engagement:**  ·Completion rate:  Control: 70.1% ; Experimental: 79.9% (p<0.001 in favor of experimental)  ·Average session performed per week:  Control: 2.06 (SD=1.16) ; Experimental: 2.09 (SD=1.03) (p<0.001 in favor of control but not meaningful)  ·Communication metrics: Average reach-outs  Control: 17.33 (SD=21.88) ; Experimental: 25.11 (SD=18.54) (p<0.001 in favor of experimental)  ·Communication metrics: Average number of messages initiated by participants  Control: 13.9 (SD=25.87) ; Experimental: 14.6 (SD=17.9) (p=0.018 in favor of experimental but not meaningful)  **Average PT-to-patient ratios:**  ·Control: 1:57 (SD=36) ; Experimental: 1:129 (SD=27) (no p value but 2.3x higher ratio in experimental)  **Average satisfaction with the program:**  ·Control: 8.60 (SD=1.76) ; Experimental: 8.76 (SD=1.75) (p<0.021 in favor of experimental but not meaningful)  **Safety:**  ·Control: 83 (0.69%) ; Experimental: 140 (0.58%) (p=0.231) |
| Chen et al. | Not clearly reported | **Average order count:**  ·Control: 331.3 (SD=5.1) ; Experimental: 326.3 (SD=23.6) (p=0.38)  **Alert suppression rate:**  ·43.7% (15,425 of 35,315 appointments)  **Average alert count:**  ·42.2% (Control: 6123.7 (SD=232.9) ; Experimental: 3541.0 (SD=669.4))  **Interacted alert count:**  ·Control: 1162.3 (SD=22.8) ; Experimental: 1118.3 (SD=71.6) (p=0.20)  **Accumulated alerts per order rate:**  ·Control: 18.5 ; Experimental: 10.9  **Accumulated interacted alerts per order rate:**  ·Control: 3.4 ; Experimental: 3.5  **Signal-to-noise ratio (using average orders as average power of signal and average count of ignored alert with no follow-up orders as average power of noise):**  ·78% (Control: 5.7% ; Experimental: 10.1%)  **Signal-to-noise ratio (using interacted alerts including follow-up orders as signal and ignored alerts with no follow-up actions as noise):**  ·97% (Control : 23.4% ; Experimental : 46.1%) |
| Cruz et al. | ·Change in adherence rate | **Adherence rate:**  ·Recommendation #1 for osteoarthritis of hip and knee (glucosamine and chondroitin)  No result  ·Recommendation #2 for osteoarthritis of hip and knee (NSAIDs)  No result  ·Recommendation #3 for anxiety (PTSD)  No result  ·Recommendation #4 for anxiety (Pregabalin)  Control: 9% ; Experimental: 16% (p=0.145)  ·Recommendation #5 for anxiety (Fluoxetine doses)  No result  ·Recommendation #6 for headache (Referral)  No result  ·Recommendation #7 for headache (Alcohol)  No result  ·Recommendation #8 for dyspepsia (Referral)  No result  ·Recommendation #9 for low back pain (Physiotherapy)  No result  ·Recommendation #10 for low back pain (SSRI)  Control: 24% ; Experimental: 100% (p=0.01 in favor of experimental)  ·Recommendation #11 for heart failure (Antialdosterones)  No result  ·Recommendation #12 for heart failure (Follow-up)  Control: 71% ; Experimental: 87% (p=0.01 in favor of experimental)  ·Recommendation #13 for urinary tract infection (Prostatitis)  Control: 44% ; Experimental: 71% (p=0.053)  ·Recommendation #14 for urinary tract infection (Fosfomycin and co-amoxiclav)  Control: 82% ; Experimental: 100% (p=0.108)  ·Recommendation #15 for urinary tract infection (Pregnant women)  Control: 50% ; Experimental: 100% (p=0.013 in favor of experimental)  ·Recommendation #16 for heart murmur (Referral)  No result  ·Recommendation #17 for osteoporosis (Calcium)  Control: 68% ; Experimental: 73% (p=0.314)  ·Recommendation #18 for osteoporosis (Biphosphonates)  Control: 84% ; Experimental: 100% (p=0.096) |
| Granviken et al. | Primary outcomes:  ·Global perceived effect  (7-point Likert scale)  ·Functional status: Clinically important improvement  (PSFS)  Secondary outcomes:  ·Pain intensity  (Numeric rating scale)  ·Functional status: Continuous scale  (PSFS)  ·Work ability  (0-10)  ·Pain self-efficacy  (PSEQ)  ·Emotional distress  (HSCL-10)  ·General musculoskeletal health  (MSK-HQ)  ·Sleep  (item from the 15D)  ·Vitality  (item from the 15D health-related quality of life questionnaire)  ·Number of pain sites  ·Use of pain medication last week  ·Number of physiotherapy treatment sessions up to maximum 12 weeks  ·Health-related quality of life  (EQ-5D) | **Global perceived effect:**  ·Week 2: OR=1.40 [0.57 ; 3.49]  ·Week 4: OR=1.59 [0.65 ; 3.91]  ·Week 8: OR=1.07 [0.44 ; 2.62]  ·Week 12: OR=1.18 [0.50 ; 2.78]  **Functional status (clinically important improvement):**  ·Week 2: OR=0.54 [0.26 ; 1.15]  ·Week 4: OR=0.65 [0.31 ; 1.37]  ·Week 8: OR=0.87 [0.41 ; 1.87]  ·Week 12: OR=0.41 [0.20 ; 0.85] (in favor of control group)  **Pain intensity:**  ·Week 12: mean difference=0.20 [-0.13 ; 0.53]  **Functional status:**  ·Week 12: Mean difference=-0.37 [-0.74 ; -0.004] (in favor of control)  **Work ability:**  ·Week 12: mean difference= -0.29 [-0.58 ; -0.03] (in favor of control)  **Pain self-efficacy:**  ·Week 12: mean difference= -0.18 [-0.52 ; 0.17]  **Emotional distress:**  ·Week 12: mean difference= -0.01 [-0.07 ; 0.05]  **General MSK health:**  ·Week 12: mean difference=0.13 [-1.68 ; 1.95]  **Number of pain sites:**  ·Week 12: mean difference=0.01 [-0.28 ; 0.31]  **Number of consultations:**  ·Week 12: mean difference=1.07 [-0.67 ; 2.81]  **Health-related quality of life:**  ·Week 12: mean difference= -0.00 [-0.03 ; 0.02]  **Sleep**  ·Week 12: OR=0.78 [0.50 ; 1.23]  **Vitality**  ·Week 12: OR=0.97 [0.59 ; 1.62]  **Use of pain medication last week:**  ·Week 12: OR=1.51 [0.96 ; 2.38]  **Treatment modalities:**  The experimental group reported using overall more treatment modalities than the control group |
| Herter et al. | Primary outcome:  ·Proportion of successful treatments.  Secondary outcome:  ·Prescription behavior of the clinicians in prescribed antibiotics  ·Number of antibiotic prescriptions | **Proportion of successful treatment:**  Within groups:  ·Control: before = 76% ; during = 76% (p=0.98)  ·Experimental: before = 75% ; during = 80% (p<0.001)  ·Experimental with certainty of AI-CDSS use: before = 75% ; during = 83% (p<0.001)  Between groups:  ·Control: 76% ; Experimental: 80% (p<0.001 in favor of experimental)  ·Subgroup analysis: Sex (p value adjusted: 0.005)  -Female: Control: 76% ; Experimental: 81% (p<0.001 in favor of experimental)  -Male: Control: 72% ; Experimental: 78% (p=0.01)  ·Subgroup analysis: Age (p value adjusted: 0.005)  -<30: Control: 81% ; Experimental 86% (p=0.01)  -30-50: Control: 80% ; Experimental: 83% (p=0.05)  -50-70: Control: 76% ; Experimental: 79% (p=0.05)  ->70: Control: 70% ; Experimental: 76% (p<0.001 in favor of experimental)  ·Subgroup analysis: Complicated urinary tract infection (p value adjusted: 0.005)  -Control: 72% ; Experimental: 79% (p=0.03)  ·Subgroup analysis: Diabetes (p value adjusted: 0.005)  -Control: 71% ; Experimental: 79% (p=0.03)  **Prescription behavior (all patients):**  Within groups:  ·Antibiotics with low tissue penetration:  Control: before = 0.79 ; during = 0.77 (p=0.02)  Experimental: before = 0.79 ; during = 0.80 (p=0.37)  -Nitrofurantoin: Control (p=0.03, decrease, not guideline-aligned) ; Experimental (p=0.56)  -Fosfomycin: Control (p=0.24) ; Experimental (p=0.70)  -Trimethoprim: Control (p=0.11) ; Experimental (p=0.58)  -Norfloxacin: Control (p=0.63) ; Experimental (p=0.001, increase but not guideline-aligned)  ·Antibiotics with high tissue penetration:  Control: before = 0.21 ; during = 0.23 (p=0.02)  Experimental: before = 0.21 ; during = 0.20 (p=0.37)  -Ciprofloxacin: Control (p=0.09) ; Experimental (p=0.04, decrease but not guideline-aligned)  -Augmentin: Control (p=0.59) ; Experimental (p=0.44)  -Sulfamethoxazole and trimethoprim: Control (p=0.005, increase but no interpretation provided) ; Experimental (p=0.65)  -Amoxicilin: Control (p=0.58) ; Experimental (p=0.45)  **Prescription behavior (certainty of AI-CDSS use):**  Between groups:  ·Antibiotics with low tissue penetration:  Control: 79% ; Experimental: 81% (p=0.15)  -Nitrofurantoin: Control: 59% ; Experimental: 53% (p<0.001 in favor of control)  -Fosfomycin: Control: 13% ; Experimental: 16% (p<0.001 in favor of experimental)  -Trimethoprim: Control: 6% ; Experimental: 8% (p=0.01 in favor of experimental)  -Norfloxacin: Control: 1% ; Experimental: 4% (p<0.001 in favor of control)  ·Antibiotics with high tissue penetration:  Control: 21% ; Experimental: 19% (p=0.15)  -Ciprofloxacin: Control:16% ; Experimental: 13% (p=0.01 in favor of control)  -Augmentin: Control: 3% ; Experimental: 3% (p=0.53)  -Sulfamethoxazole and trimethoprim: Control: 2% ; Experimental: 2% (p=0.39)  -Amoxicilin: Control: 1% ; Experimental: 1% (p=0.02, not able to interpret)  **Number of antibiotic prescriptions**  Within groups:  ·Control: before = 5044 ; during = 3360  ·Experimental: before = 4998 ; during = 3422 |
| Liu et al. | ·Diagnostic accuracy  ·Rate of attendance at ophthalmology appointments | **Diagnostic accuracy:**  ·Sensitivity: 100% [92.3 ; 100]  ·Specificity: 65.7% [57.0 ; 73.7]  ·Positive predictive value: 50%  ·Negative predictive value: 100%  **Rate of attendance at ophthalmology appointments:**  ·At 3 months:  Control: 9.4% ; Experimental: 32.6% (p<0.0001 in favor of experimental)  ·At 6 months:  Control: 13.4% ; Experimental: 46.7% (p<0.0001 in favor of experimental)  ·At 12 months:  Control: 18.7% ; Experimental: 55.4% (p<0.0001 in favor of experimental)  Subgroups analysis (only experimental): rate of attendance based on the screening result (p=0.75)  -Referable diabetic eye disease: 46.7%  -Vision-threatening diabetic eye disease: 58.3%  -Inconclusive screening: 56.6% |
| Popescu et al.  Qassim et al. | ·Appointment length  ·Physician-patient relationship  (STAR-P for patients and STAR-C for clinicians)  ·Perceived clinical utility  ·Trust in the AI-CDSS  ·Usability of the AI-CDSS  ·Engagement with the app  ·Side effects  (PRISE-20; FIBSER; UKU scale) | **Average appointment length (in minutes):**  ·Control: 19.53 (SD=6.09) ; Experimental: visit 1 = 17.69 (SD=10.12) and visit 2 = 21.48 (SD=10.69) (p=0.46)  **Physician-patient relationship:**  ·STAR-P:  Mean total score:  Pre: 38.4 (SD=12)  Post: 42.69 (SD=5.57)  Positive clinical input:  Pre: 9.3 (SD=3.0)  Post: 10.23 (SD=1.88)  Positive collaboration:  Pre: 19.9 (SD=6.7)  Post: 21.92 (SD=2.24)  Non-supportive clinical input:  Pre: 9.3 (SD=3.3)  Post: 10.54 (SD=2.58)  ·STAR-C:  Mean total score:  Pre: 31.5 (SD=6.9)  Post: 40.29 (SD=5.65)  Positive clinician input:  Pre: 8.9 (SD=1.6)  Post: 9.93 (SD=1.38)  Positive collaboration:  Pre: 7.4 (SD=2.7)  Post: 17.0 (SD=2.60)  Emotional difficulties:  Pre: 15.3 (SD=4.0)  Post: 10.57 (SD=1.79)  ·46% of the patients felt that the app significantly or somewhat improved the patient-physician relationship  **Perceived clinical utility:**  Clinicians:  ·86% reported the AI-CDSS was useful for getting a better sense of their patients’ situations  ·86% thought the AI/predictive model was useful in helping them make their treatment decisions  ·71% felt the information provided by the tool was helpful  ·71% believed they would continue to use this application after the study  ·57% would use the AI/predictive model in future clinical practice  Patients:  ·77% reported keeping track of their symptoms and treatments in the tool helped their physician better manage their care.  ·62% felt the tool allowed them to fully report how they were feeling  ·62% reported the questions covered the things most important to them  ·62% felt the app improved their care  ·0% noted that their care worsened as a result of the tool  **Trust:**  Clinicians:  ·71% trusted the tool  Patients:  ·62% trusted the tool  **Usability:**  Clinicians:  ·71% rates AI-CDSS easy to use  Patients:  ·92% rated AI-CDSS easy to use  **Engagement:**  Clinicians:  ·100% logged into the AI-CDSS at visit 1 and visit 2  ·93% used the clinical algorithm module at visit 1 and visit 2  Patients:  ·Mean completion rate of PHQ-9: 59% (SD=28%)  ·Mean completion rate of GAD-7: 60% (SD=28%)  ·71% completed ≥50% of PHQ-9/GAD-7 assessments  **Side effects:**  ·12% experienced side effects  ·No serious adverse events related to the tool |
| Romero-Brufau et al. | Not clearly reported | Clinician attitudes:  **Care coordination:**  ·Control: 11 favorable vs. 14 unfavorable ; Experimental: 21 favorable vs. 3 unfavorable (p<0.01 in favor of experimental)  **Patient preparation for managing diabetes:**  ·Control: 19 favorable vs. 8 unfavorable ; Experimental: 26 favorable vs. 2 unfavorable (p=0.04 in favor of experimental)  **Reducing complications:**  ·No significant difference  **Intervention effectiveness:**  ·No significant difference  **Right intervention:**  ·No significant difference  **Familiarity with AI:**  ·Control: 17 favorable vs. 15 unfavorable ; Experimental: 24 favorable vs. 1 unfavorable (p<0.01 in favor of experimental)  **Routinely use AI:**  ·No significant difference  **Excited about AI:**  ·Control: 28 favorable vs. 3 unfavorable ; Experimental: 11 favorable vs. 9 unfavorable (p<0.01 in favor of control)  **AI will complicate my job:**  ·No significant difference  **AI will make my job obsolete:**  ·No significant difference  **AI does not understand my job:**  ·Control: 16 favorable vs. 9 unfavorable ; Experimental: 3 favorable vs. 17 unfavorable (p<0.01 in favor of experimental)  **Users’ ability to identify the right intervention:**  ·median= 11/100  ·34% felt that the system had the potential to be useful with improvements  ·14% would recommend the AI-CDSS to another clinic  ·10% felt that the AI-CDSS should continue to be integrated in the clinical |
| Seol et al. | Primary outcome:  ·Occurrence of asthma exacerbation within 1 year  Secondary outcomes:  ·Clinicians’ burden for reviewing and collecting clinical data from EHRs for making a clinical decision  ·Clinicians’ satisfaction  ·Health care cost  ·Asthma control status  (ACT or Childhood ACT or TRACK test)  ·Timeliness of asthma follow-up care after asthma exacerbation  ·Enrollment in an asthma management program  ·Adverse events | **Occurrence of asthma exacerbation:**  ·Control: 15% ; Experimental: 12% (OR=0.82 [0.374 ; 1.96], p=0.626)  **Clinicians’ burden (in minutes):**  ·Control: median = 11.3 [IQR 6.3 ; 15] ; Experimental: median = 3.5 [IQR 2 ; 5] (p<0.001 in favor of the experimental)  **Clinicians’ satisfaction for AI-CDSS:**  ·median= 7/10  **Health care costs:**  ·Control: +80$ [-841 ; 1000] ; Experimental: -1036$ [-2177 ; 44] (p=0.12)  **Asthma control status (% time with well-controlled asthma):**  ·Control: median = 100% [IQR 100 ; 100] ; Experimental: median = 100% [IQR 75-100] (p=0.56)  **Timeliness of follow-up after exacerbation (in days):**  ·Control: median = 28 days [IQR 6 ; 48] ; Experimental: median = 7 [IQR 2 ; 27] (HR=1.93 [0.82 ; 4.45], p=0.10)  **Enrollment in an asthma management program:**  ·Control: 0 ; Experimental: 8  **Adverse events:**  ·0 serious adverse events resulting in death or breach of confidentiality in both groups |
| Rushlow et al.  Thao et al.  Yao et al. | ·Newly diagnosed low ejection fraction  ·Impact of adoption on the clinical outcome of low ejection fraction. Adoption of AI was defined at the clinician level by dividing the number of echocardiograms for AI positive ECGs by the total number of AI-positive ECGs. Each clinician was then categorized into either the high adopter group (top quartile) or low adopter group (lower three quartiles).  ·Cost-effectiveness | **Newly diagnosed low ejection fraction:**  ·Overall cohort:  Control: 1.6% ; Experimental: 2.1% (OR=1.32 [1.08 ; 1.61], p=0.007 in favor of experimental)  ·Among patients with a high likelihood of low ejection fraction:  Control: 14.5% ; Experimental: 19.5% (OR=1.43 [1.08 ; 1.91], p=0.01 in favor of experimental)  ·Subgroup analysis : Outpatient ECG  Control: 1.0% ; Experimental: 1.6% (OR=1.71 [1.23 ; 2.37], p=0.001 in favor of experimental)  **Echocardiography utilization:**  ·Overall cohort:  Control: 18.2% ; Experimental: 19.2% (OR=1.08 [0.97 ; 1.20], p=0.17)  ·Among patients with a high likelihood of low ejection fraction:  Control: 38.1% ; Experimental: 49.6% (OR=1.63 [1.24 ; 2.13], p<0.001 in favor of experimental)  **Impact of adoption (only experimental):**  ·Echocardiograms ordered after positive AI result:  Low adopters: 43.0% ; High adopters: 78.7%  ·Of the echocardiograms ordered:  Low adopters: 37.9% ; High adopters: 43.0% showed low ejection fraction  ·Diagnostic yield (proportion of AI-positive with confirmed ejection fraction)  Low adopters: 16.3% ; High adopters: 33.9% (p<0.001 in favor of high adopters)  · Echocardiograms ordered after negative AI result:  Low adopters: 17.1% ; High adopters: 18.0%  ·Of the echocardiograms ordered:  Low adopters: 5.7% ; High adopters: 6.4% showed low ejection fraction  ·Diagnostic yield (proportion of AI-positive with confirmed ejection fraction)  Low adopters: 1.15% ; High adopters: 0.97% (p=0.46)  ·Likelihood of detecting low ejection (adjusted):  OR=1.62 [1.21 ; 2.17], in favor of high adopters  **Cost-effectiveness (with a 100,000$ threshold):**  ·Base-case analysis:  Cost (in $): Control: 224,564 ; Experimental: 224,950  LYs: Control: 19.72 ; Experimental: 19.73  QALYs: Control: 14.83 ; Experimental: 14.85  ICER ($/QALY): 27,858 => cost-effective  ·Scenario analysis: Age  20 years old: ICER: 13,007 => cost-effective  40 years old: ICER: 20,419 => cost-effective  60 years old: ICER: 27,713 => cost-effective  70 years old: ICER: 34,196 => cost-effective  80 years old: ICER: 36,213 => cost-effective  ·Scenario analysis: Time frame  10 years: ICER: 35,698 => cost-effective  20 years: ICER: 30,095 => cost-effective  50 years: ICER: 27,858 => cost-effective  ·Scenario analysis: AI-positive only patients  ICER: 23,435 => cost-effective  ·Scenario analysis: Patients with underlying low ejection fraction  ICER: 13,136 => cost-effective  ·Scenario analysis: Outpatient  ICER: 1,651 => cost-effective  ·Uncertainty  Probability of being cost-effective at 100,000$/QALY: 79.5% |

***15-D***: 15-Dimensional health-related quality of life; ***AI-CDSS***: Artificial Intelligence-enhanced Clinical Decision Support System; ***ACT***: Asthma Control Test; ***ECG***: Electrocardiography; ***EHS***: Emergency Health Services; ***EQ-5D***: European Quality of Life 5 Dimensions; ***FIBSER***: Frequency, Intensity, and Burden of Side Effects Rating scale; ***GAD-7***: Generalized Anxiety Disorder-7 items; ***HSCL-10***: Hopkins Symptoms Checklist-10 items; ***ICER***: Incremental Cost-Effectiveness Ratio; ***LYs***: Life-Years; ***MSK-HQ***: Musculoskeletal Health Questionnaire; ***NPRS***: Numeric Pain Rating Scale; ***NSAIDs:*** NonSteroidal Anti-Inflammatory Drugs; ***OR***: Odds ratio; ***PHQ-9***: Patient Health Questionnaire-9 items; ***PRISE-20***: Patient-Rated Inventory of Side Effects-20 items; ***PSEQ***: Pain Self-Efficacy Questionnaire; ***PSFS***: Patient-Specific Functional Scale; ***PT:*** Physiotherapist; ***PTSD***: Post-Traumatic Stress Disorder; ***QALYs***: Quality-Adjusted Life Year; ***SD***: Standard Deviation; ***SSRIs***: Selective Serotonin Reuptake Inhibitors; ***STAR-C***: Scale To Assess therapeutic Relationships-Clinician version; ***STAR-P***: Scale To Assess therapeutic Relationships-Patient version; ***TRACK test***: Test for Respiratory and Asthma Control in Kids test; ***UKU-Scale***: Udvalg for Kliniske Undersøgelser side effects rating scale
