# Supplementary material for: AI-Based Clinical Decision Support Systems in Primary Care: Systematic Review of Clinical Implementation
Source: J Med Internet Res. 2026 Oct 19;28:e96882. doi: 10.2196/96882 (PMC13679871; doi:10.2196/96882)
Supplement: Supplementary file 12 [file jmir-v28-e96882-s006.docx]

**Multimedia appendix 9: Risk of bias assessment**
